# Adaptation of the Mitochondrial Genome in Cephalopods: Enhancing Proton Translocation Channels and the Subunit Interactions

**DOI:** 10.1371/journal.pone.0135405

**Published:** 2015-08-18

**Authors:** Daniela Almeida, Emanuel Maldonado, Vitor Vasconcelos, Agostinho Antunes

**Affiliations:** 1 CIIMAR/CIMAR, Interdisciplinary Centre of Marine and Environmental Research, University of Porto, Porto, Portugal; 2 Department of Biology, Faculty of Sciences, University of Porto, Porto, Portugal; University of Connecticut, UNITED STATES

## Abstract

Mitochondrial protein-coding genes (mt genes) encode subunits forming complexes of crucial cellular pathways, including those involved in the vital process of oxidative phosphorylation (OXPHOS). Despite the vital role of the mitochondrial genome (mt genome) in the survival of organisms, little is known with respect to its adaptive implications within marine invertebrates. The molluscan Class Cephalopoda is represented by a marine group of species known to occupy contrasting environments ranging from the intertidal to the deep sea, having distinct metabolic requirements, varied body shapes and highly advanced visual and nervous systems that make them highly competitive and successful worldwide predators. Thus, cephalopods are valuable models for testing natural selection acting on their mitochondrial subunits (mt subunits). Here, we used concatenated mt genes from 17 fully sequenced mt genomes of diverse cephalopod species to generate a robust mitochondrial phylogeny for the Class Cephalopoda. We followed an integrative approach considering several branches of interest–covering cephalopods with distinct morphologies, metabolic rates and habitats–to identify sites under positive selection and localize them in the respective protein alignment and/or tridimensional structure of the mt subunits. Our results revealed significant adaptive variation in several mt subunits involved in the energy production pathway of cephalopods: ND5 and ND6 from Complex I, CYTB from Complex III, COX2 and COX3 from Complex IV, and in ATP8 from Complex V. Furthermore, we identified relevant sites involved in protein-interactions, lining proton translocation channels, as well as disease/deficiencies related sites in the aforementioned complexes. A particular case, revealed by this study, is the involvement of some positively selected sites, found in Octopoda lineage in lining proton translocation channels (site 74 from ND5) and in interactions between subunits (site 507 from ND5) of Complex I.

## Introduction

The mitochondrial subunits of the respiratory chain complexes (mt subunits)–cytochrome c oxidase subunit 1–3 (COX1, COX2, COX3), cytochrome b (CYTB), NADH dehydrogenase subunit 1–6 (ND1, ND2, ND3, ND4, ND5, ND6), NADH dehydrogenase subunit 4L (ND4L), ATPase F0 subunit 6 (ATP6) and ATPase F0 subunit 8 (ATP8)–encoded by 13 mitochondrial genes (mt genes) in most metazoans, are involved in several key evolutionary processes of eukaryotes having a major role in the production of energy [1]. The mt subunits interact with nuclear-encoded proteins establishing four of the five multi-subunit enzyme complexes–Complex I (ND1, ND2, ND3, ND4, ND4L, ND5 and ND6), III (CYTB), IV (COX1, COX2 and COX3) and V (ATP6 and ATP8)–involved in the respiratory chain of aerobic cells. Complex II consists entirely of nuclear-encoded proteins. Globally, Complexes I, II, III and IV are responsible for the mitochondrial oxidative phosphorylation (OXPHOS) pathway and the Complex V uses the generated energy gradient to synthesize adenosine triphosphate (ATP) [2].

The effect of any amino acid substitution can vary greatly depending on its location in the protein structure. Thus, site-specific amino acid substitutions that do not confer structural changes in the protein can affect protein function, such as the changes in binding sites. Some amino acid changes in mt subunits cause inefficiencies in the electron transfer chain system, contributing to the increase of reactive oxygen species (ROS) that can lead to the disruption of OXPHOS [3–5]. On the other hand, the amino acid substitutions can also improve or decrease aerobic capacity and be linked with life history traits and environmental adaptation [6–8].

Under strong selective pressure, organisms may evolve adaptations to increase their survival rate under certain ecological conditions. This can be achieved by changes in protein structure through amino acid substitutions, and/or by altering the amount of protein expression through modifications in the transcriptional regulation [9]. Dowling and colleagues (2008) highlighted the key importance of the mitochondrial-nuclear interaction as a unit of selection and the consequences of mitochondrial encoded fitness effects on several key evolutionary processes [2]. Furthermore, mt genes have been proposed to be connected with species adaptation to altitude [10] and temperature [11]. However, most of the studies on mt gene mutations that affect phenotypic variation, with positive selection shaping their evolution, have been conducted on vertebrates (mammals [5, 12] and fish [13]). Thus, there is little information with respect to the adaptation of the mt genomes of invertebrates, particularly marine invertebrates.

Cephalopoda (octopus, squid, cuttlefish, *Nautilus*) is the Class of the Mollusca phylum with the third highest number of species (~ 700 extant species) presenting remarkable morphological and physiological innovations [14]. Overtime, these marine invertebrate species have evolved several biological features such as a highly advanced visual and nervous system, camouflage strategies, diversified body shapes, sizes and metabolic rates, making them highly competitive and efficient predators, which contribute to their successful worldwide dispersion [14]. Thus, cephalopods cover a wide range of marine depths, some species are found from the sea surface to 100m in depth (*Sepioteuthis lessoniana*) [15] and others between 600 to 1200m in depth (*Vampyroteuthis infernalis*) [16].

All these features make cephalopods an attractive group of species to study signatures of selection of the mitochondrial-encoded proteins. Therefore, we consider 17 mt genomes from cephalopod species–representing eight major taxonomic groups from the Class Cephalopoda–to evaluate the role of selection in the mt genes that may affect the adaptive features of cephalopods. We perform detailed molecular evolution analyses–in particular, branch [17, 18] and branch-site [19] specific likelihood analyses–to test several branches of interest related with species habitats (distinct depths), metabolic rates and physical traits (number of arms), where positive selection may be significantly influencing the evolution of cephalopod mt genes. We identify the location of positively selected sites in the Cephalopoda protein sequence alignments. Then, we analyze the amino acid physicochemical properties of these sites and discuss them in the context of well-studied respiratory chain complexes (through structure-based homology comparisons), in order to assess whether these properties might have changed in functionally important regions.

## Materials and Methods

### Sequence collection

The complete coding-sequences (CDS) of the 13-mt genes from 17 fully sequenced cephalopod mt genomes, covering the major taxonomic groups Oegopsida, Myopsida, Bathyteuthoidea, Sepiolidae, Sepiidae, Vampyromorpha, Octopoda and Nautiloidea (S1 Table), have been retrieved from GENBANK [20] and MITOZOA [21–23] databases.

### Alignment and phylogenetic analyses

Since some Cephalopoda mt genomes have duplicated protein-coding genes (S2 Table)–a feature only found in Oegopsida and Bathyteuthoidea species (S2 Table) [24, 25]: with *cox1*, *cox2*, *atp6* and *atp8* genes duplicated in *Dosidicus gigas* (NC_009734), *Sthenoteuthis oualaniensis* (EU660577), *Todarodes pacificus* (NC_006354), *Watasenia scintillans* (NC_007893), *Architeuthis dux* (FJ429092) and *Bathyteuthis abyssicola* (AP012225) and *cox3* that is also duplicated in all the above species except for *Bathyteuthis abyssicola* (S2 Table)–the codon based CDS alignment of the aforementioned genes allowed the verification that the duplicated genes are highly conserved; some are exact copies and others present slight nucleotide substitutions (S2 Table).

Thus, in order to find the best way to deal with duplicated genes to assess the phylogenetic relationships among the species of the Class Cephalopoda, we considered two datasets: one of them with 13 protein-coding genes where a sequence of a single copy of duplicated genes was included (the left side duplicated genes; see S1 Fig) and other containing 13 protein-coding genes with the other copy of duplicated genes (the right side duplicated genes; see S1 Fig). For each one of the mentioned datasets, individual codon based CDS alignments of the mt genes were performed using MUSCLE [26] integrated in the SEAVIEW software version 4.4.0 [27]. All the alignments were manually inspected and concatenated in GENEIOUS software version 5.6.7 [28].

The substitution nucleotide model that best fit each one of the Cephalopoda datasets was calculated using the Akaike Information Criterion (AIC), implemented in JMODELTEST software version 2.1.1 [29], starting with 11 substitution schemes and using the fixed BIONJ-JC option base tree for likelihood calculations. For both datasets, the selected model was the General Time Reversible (GTR) model, with a proportion of invariable sites (I) and heterogeneity of substitution rates among sites modeled following a gamma distribution (G). Additionally, we performed the Xia *et al*. statistic test [30], implemented in the DAMBE software version 5.3.31 [31], on each codon position of our datasets, in order to check for evidences of saturation bias that could derail phylogenetic/adaptive analyses. This test compares the saturation index expected when assuming full saturation (ISS.c, critic value) with the observed saturation index (ISS), assuming symmetrical (Iss.c Sym) and asymmetrical (Iss.c Asym) topologies, which did not showed significant evidence of saturation (*p* < 0.05) at the first and second codon positions. For the third codon positions, this test suggested that they are only marginally useful for phylogenetic reconstruction when the true tree topology is symmetrical and useless if the true tree topology is asymmetrical [30].

For each dataset, phylogenies were estimated using the Maximum likelihood (ML) and Bayesian (BA) inference methods. ML phylogenetic trees were built in PHYML software version 3.0 [32], with 1000 bootstrap replicates and the parameter ‘best of Nearest Neighbour Interchange (NNI) and Subtree Pruning and Regrafting (SPR)’ branch search algorithms. BA trees were inferred in MRBAYES software version 3.2 [33]. Two runs with two chains of the Markov Chain Monte Carlo (MCMC) method were performed for 10000000 generations, with sampling trees every 100 generations. The following criteria were used to test convergence of the chains: the standard deviation of split frequencies was less than 0.01 and the potential scale reduction factors (PSRF) were close to 1.0 for all parameters. Additionally, we used TRACER software version 1.6 [34] to assess if the chains have converged to its stationary distribution for all the parameters, and to better determine the appropriate burn-in (2500000 of the sampled trees were discarded). The effective sample size (ESS) for all the parameters was greater than 800 (ESS for the log-likelihood = 4232).

To assess the effect of possible saturation biases at the third codon position nucleotides on tree topology, we created modified datasets (RY coded alignments) in which all third codon positions were coded as purines (adenine and guanine: R) or pyrimidines (cytosine and thymine: Y) [35–38]. The RY coding of the alignments were performed according to Meiklejohn et al. (2014) [37]. These RY coded alignments were analyzed under the same parameters previously described for ML phylogenetic analyses.

### Selection analyses

The effect of natural selection on the evolution of the 13-mt genes was assessed by comparing the number of non-synonymous substitutions per non-synonymous sites (d_N_) with that of synonymous substitutions per synonymous sites (d_S_), where negative/purifying selection is characterized by a d_N_/d_S_ (ω) ratio < 1 and positive/diversifying selection by a ω > 1, using: site, branch and branch-site specific likelihood analyses. For all the selection analyses the *atp6*, *atp8*, *cytb*, *cox1*, *cox2*, *cox3*, *nd1*, *nd2*, *nd3*, *nd4*, *nd4l*, *nd5* and *nd6* individual alignments and the cephalopod tree topology (unrooted), previously obtained in this study, were submitted to the CODEML program from the PAML 4.7 package [39, 40].

Site models use a statistical distribution to account for variation of the ω ratio among sites (codons) [41, 42]. In these analyses likelihood ratio tests (LRTs) compare the maximum likelihoods of a null model that does not allow for any codons with ω > 1 (M0, M1a, M7, M8a) against an alternative model that allows sites with ω > 1 (M3, M2a, M8) [40], to find which model fits the data significantly better. LRTs were conducted by comparing the following nested models: M0 (one ratio: assumes a constant ω ratio for all sites) *vs*. M3 (discrete: assumes 3 site classes for ω ratio estimated from the data) [43–46], M1a (nearly neutral: one ω class between 0 and 1, and one class of ω = 1) *vs*. M2a (positive selection: same as M1a model plus an extra class of ω > 1) [41, 47, 48], M7 (beta: assumes a beta distribution for 0 ≤ ω ≥ 1) *vs*. M8 (beta&ω: same as the M7 model plus an extra class of ω > 1) [42] and M8a (beta&ω = 1: same as M7 plus an extra class of ω = 1) *vs*. M8 (beta&ω) [49]. Positive selection was indicated when a freely estimated ω parameter was greater than 1 and the LRT was significant.

Branch models test for different evolutionary rates in specific branches of the phylogeny [17, 18], comparing the average of foreground ω values (branches of interest, ϖ_F_) with the average of background ω values (other branches, ϖ_B_), using as null hypothesis ϖ_F_ = ϖ_B_ and alternative hypothesis ϖ_F_ ≠ ϖ_B_. Following this test, if the alternative (unconstrained) model was found as a better fit to the data, then it needs to be tested against another null (constrained) model where ϖ_F_ = 1, in order to check for the prevalence of the previous selection result.

The branch-site specific likelihood analyses (modified branch-site model A) test for episodic positive selection on particular residues along the foreground-lineages [19], allowing ω to vary, both, among sites in the protein and across branches on the tree. For this test, the alternative model assumes that only the foreground-lineages (branches of interest) may have experienced positive selection, admitting three ω_F_ values (0 < ω_F_ < 1, ω_F_ = 1 and ω_F_ > 1), and the null model constraints ω_F_ = 1.

In branch and branch-site specific likelihood analyses the branches of the phylogenetic tree are divided *a priori* into foreground- (branch of interest) and background-branches (the remaining branches). In this study we tested if there are positive selection in any of 8 foreground-branches (named A to H). These analyses consider only one foreground branch at a time, thus we performed 8 analyses employing branch and branch-site approaches. More specifically, we tested for positive selection in: (A) genes of species living in deep waters; (B) squids that present high metabolic rates; (C) the Loliginidae family, having the highest metabolic rates relatively to all other lineages of the tree; (D) the Sepiidae group, with high metabolic rate and species living in shallow waters; (E) Octopoda, which has intermediate metabolic rates and species living in shallow waters; (F) Decapodiformes, cephalopods with 10 arms and high metabolic rates; (G) Octopodiformes, cephalopods with 8 arms and low or intermediate metabolic rates; (H) *Nautilus macromphalus*, with low metabolic rate and dwelling in deep. S1 Table contains more details about the species features mentioned here.

For all these analyses we applied the F3x4 codon model [50] allowing for ML estimation of κ (transition/transversion ratio) and ω. All the models were run several times, adjusting the initial κ and ω values in order to avoid local optima. For all the model comparisons, the hypothesis decision was calculated as the double of the difference between the alternative and null model log likelihood (2ΔlnL) and assuming that the null distribution of these results can be approximated by a chi-square (χ^2^) distribution (p-value < 0.05). The number of degrees of freedom (df) was calculated as the difference of the number of estimated parameters between the nested models [42, 47]. Finally, when it was revealed that the alternative model fit the data better, the codon sites under positive selection were identified using the Bayes Empirical Bayes (BEB) calculation, which analyzes the posterior probabilities for these sites [48].

We also used the DATAMONKEY web server [51, 52] to assess codons under purifying selection [40, 48], implementing the fixed effects likelihood model (FEL, site-by-site analysis) [53] and to obtain further evidences of episodic diversifying selection employing the mixed effects model of evolution (MEME; branch-site analysis) [54], both inferred at p-value < 0.05 significance level. FEL model compares the instantaneous d_S_ and d_N_, based on a codon-substitution model, without assuming a prior d_N_/d_S_ [53]. This model was chosen since it is a rigorous method and tends to be less conservative and to have less false positives/negatives than other methods in datasets of intermediate size [52]. MEME model is capable of identifying instances of both, episodic and pervasive positive selection allowing the distribution of ω to vary across lineages at individual sites [54].

TREESAAP software [55]–designed to classify the impact of amino acid replacements on local physicochemical properties in eight magnitude categories, from the most conservative (1) to the most radical (8) [56, 57]–was employed to infer about the strong positive selection for a given physicochemical amino acid property. Thus, only changes of great magnitude (7 and 8 categories) at the p-value < 0.001 (z-score > |3.09|) significance level were considered.

### Protein structure analyses

In order to understand how the sites under positive selection, influence the structure and/or function of the mt subunits, we located them in the respective Cephalopoda protein sequence alignment. To perform this, we used the Alignment Filter tool of the IMPACT_S software [58]. These sites were identified (p-value < 0.05) by the BEB (CODEML branch-site model test) and MEME (DATAMONKEY episodic diversifying selection) analyses. We also mapped and analyzed the results displayed by the TREESAAP analyses (p-value < 0.001), for the same mt subunits, in order to get additional insight about the amino acid properties of common sites, identified by the previous methodological approaches.

Furthermore, to complement our analyses with the spatial position of these sites in a tridimensional plane, and since the X-ray crystal structures of the mitochondrial encoded proteins from cephalopods are not available on the Protein Data Bank (PDB) [59], we modeled its 3D structures for the *Octopus vulgaris* species, from ND5 (YP_112440.1), ND6 (YP_112444.1), CYTB (YP_112443.1), COX2 (YP_112437.1), COX3 (YP_112433.1) and ATP8 (YP_112438.1), using the I-TASSER server with default parameters [60]. This species was chosen due to its worldwide dispersion and because it is one of the cephalopod species widely studied and known by most of the world population. We have only modeled the 3D structure of proteins for which evidence of positive selection was detected. I-TASSER is a platform that allows the automatic generation of high-quality predictions of 3D structure and biological function of proteins from their amino acid sequences, using PDB templates or by *ab initio* modeling approaches, where the assembly follows several Monte Carlo simulations. The quality of the generated model is evaluated considering a confidence score (C-score) value, which is calculated based on the significance of threading template alignments and the convergence parameters of the structure assembly simulations [60]. Also a score scale is attributed to the structural similarity between two structures (T-score) in order to guarantee that the resulting topology is not random. Following the author instructions to evaluate the reported scores [60], the most likely structure for each of the six mt subunits modeled was retrieved from I-TASSER. Then, we performed the superimposition of available X-ray crystal structures–ND5 (*Escherichia coli* PDB: 3RKO), CYTB (*Bos taurus* PDB: 1PPJ and *Saccharomyces cerevisiae* PDB: 1P84), COX2 (*Bos taurus* PDB: 1V54), COX3 (*Rhodobacter sphaeroides* PDB: 1M56)–with their corresponding 3D structures of *Octopus vulgaris*, in which the positively selected sites were mapped. The superimposition, visualization and manipulation of the 3D structures were performed with PYMOL software version 1.5.0.4 [61]. When, the obtainment of a reliable 3D model was not satisfactory, which were the cases of the ND6 and ATP8, insight into the evolution of the mt subunits in cephalopods was obtained by inspection of the amino acid substitutions in their protein sequence alignments. Additional information about the topology and orientation of the transmembrane (TM) domains of these subunits was obtained using the TMHMM server version 2.0, which is a membrane protein topology prediction method based on a hidden Markov model [62].

Since the mt subunits of the respiratory chain complexes are highly conserved among species (sharing the same cofactors and performing the same functions) [13], we searched if the positively selected sites, present in the sequence alignments of cephalopods, are related with important functional and/or mutational sites reported in the literature (*e*.*g*: *Homo sapiens*, *Escherichia coli*, *Bos taurus*, *Saccharomyces cerevisiae* and *Rhodobacter sphaeroides*) [63–83]. Therefore, through profile alignments (using GENEIOUS software version 5.6.7 [28]), we performed homology analyses of the mt subunits from our Cephalopoda datasets with their well-studied counterparts in other species. The details of these analyses for each one of the mt subunits, with positively selected sites, are described in the following tables: ND5 (S3 Table), ND6 (S4 Table), CYTB (S5 Table), COX2 (S6 Table) and COX3 (S7 Table).

## Results

### Phylogenetic analyses

**Fig 1:**
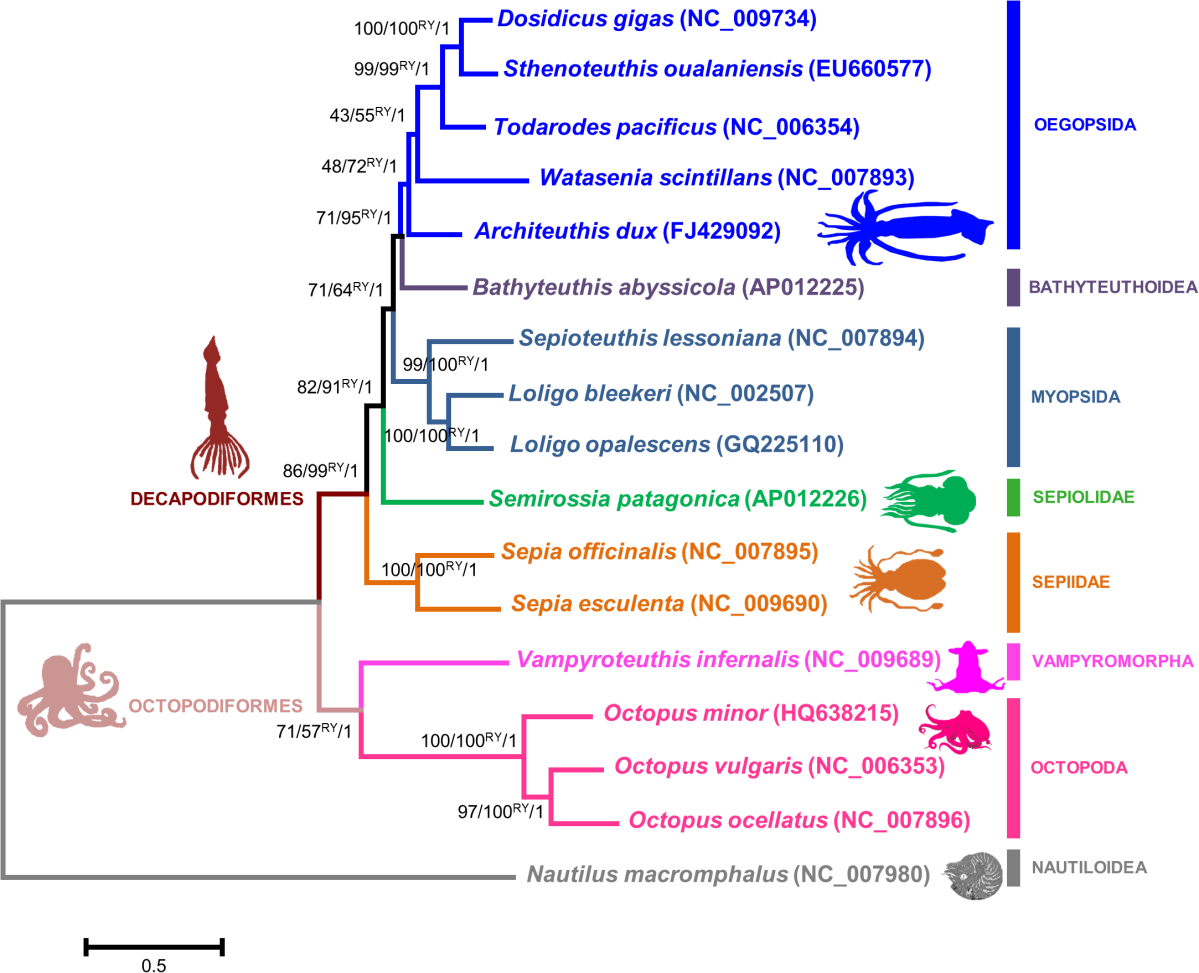
Cephalopoda mitogenomic consensus phylogenetic tree. The topology shown corresponds to the Maximum likelihood (ML) tree. The bootstrap probabilities (%) supporting each node were estimated with ML analyses using PHYML software version 3.0 (1000 bootstrap replicates, GTR+G+I model and best of NNI&SPR branch search algorithm) and are shown by the first and second values next to the branches. The RY symbol indicates that these values resulted from the RY coded alignments. The posterior probability supporting each node was estimated from Bayesian analyses using MRBAYES (GTR+G+I model; 10 000 000 generations; a sample frequency of 100 and a burn-in corresponding to 25% of the sampled trees) and corresponds to the third value next to the branches. The tree is drawn to scale, with branch lengths measured in the number of substitutions per site. Superorders–Decapodiformes (10 arms) and Octopodiformes (8 arms). The species are designated by their scientific names followed by the respective accession numbers. On the right, are indicated the names of the major taxonomic groups of cephalopods.

Regardless of the dataset used (with either copy of duplicated genes and their RY coded versions), the Cephalopoda tree topology obtained was the same (Fig 1). The ML and BA analyses for the 17 cephalopods consistently supported the Decapodiformes clade, with bootstrap percentages (BP) of 86% (from the original alignments), 99% from the RY coded alignments (99%RY) and Bayesian posterior probability (PP) of 1 (Fig 1). With respect to the intra-relationships of Decapodiformes, these analyses strongly supported a close relationship between the Oegopsida species *Dosidicus gigas*, *Sthenoteuthis oualaniensis* and *Todarodes pacificus* (BP of 99%, BP of 99%RY and PP of 1), contrasting with the relationships of the species *Watasenia scintillans* and *Architeuthis dux* that are not strongly supported (Fig 1).

Moreover, these analyses also revealed a high support to the species relationships within the Myopsida clade (BP of 99%, BP of 100%RY and PP of 1), as well as, to the sister relationship of Sepiidae to all other Decapodiformes (BP of 86%, BP of 99%RY and PP of 1) (Fig 1). The topology obtained also suggested that *Semirossia patagonica* is sister taxon to the clade containing Myopsida, Bathyteuthoidea and Oegopsida (BP of 82%, BP of 91%RY and PP of 1) (Fig 1). Furthermore, the Octopoda clade was highly supported (BP of 100%, BP of 100%RY and PP of 1) and the monophyly of Octopoda and Vampyromorpha was suggested (Fig 1).

### Purifying selection in Cephalopoda mitochondrial genes

**Fig 2:**
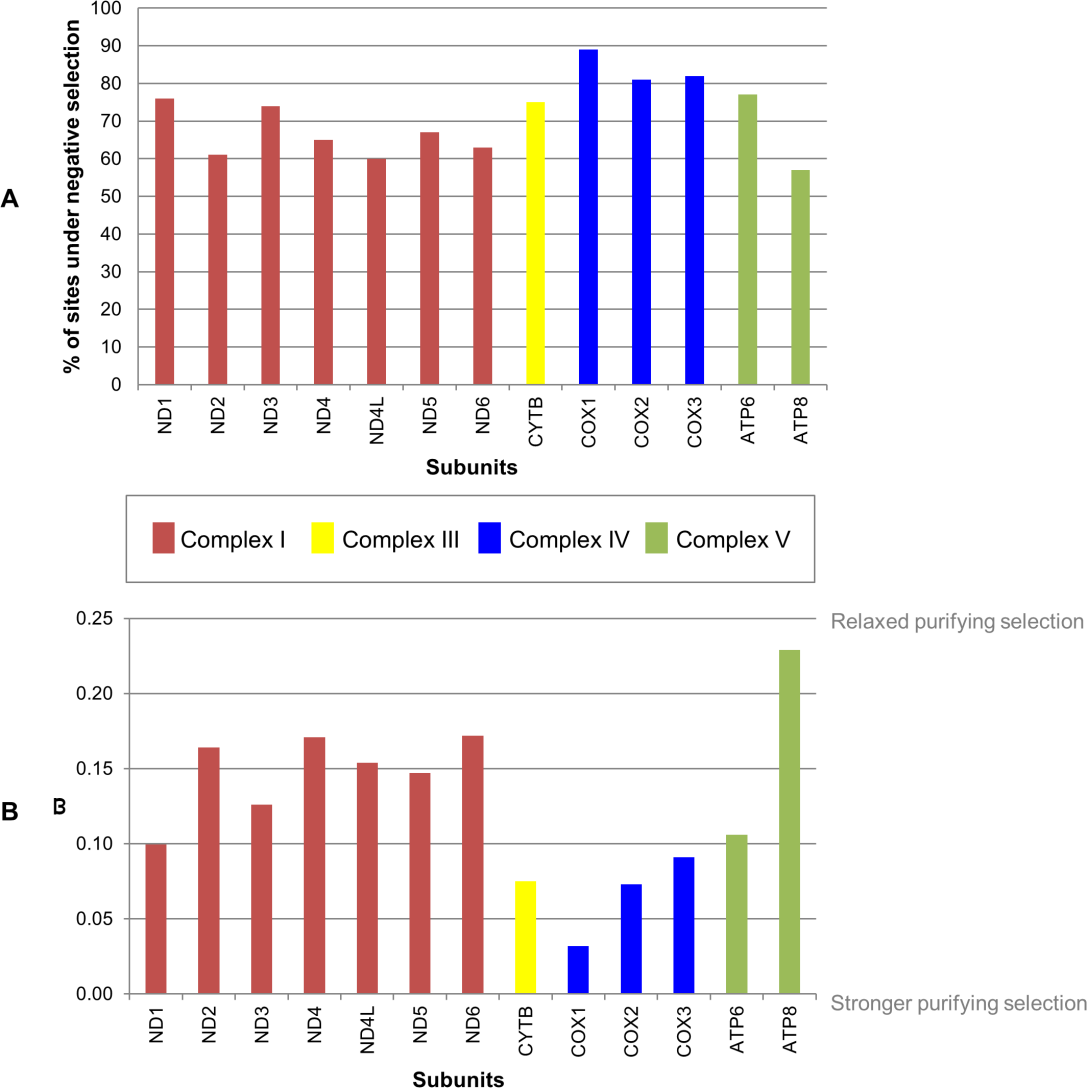
Negative selection reported by FEL site-by-site analyses. (A) Percentage of codons under negative selection in cephalopod mitochondrial subunits. (B) Cephalopoda ϖ estimates for each one of the mitochondrial subunits.

Employing site-specific analyses–site models (CODEML) and FEL analyses (DATAMONKEY web server)–we were able to infer the global molecular evolutionary rates acting in each one of the 13-mt genes of our Cephalopoda dataset. In the site model analyses from CODEML, for all the mt genes, the null model was rejected only in the M0 *vs*. M3 test (p-value < 0.05) (S8 Table). This means that for all the analyzed mt genes, the ϖ ratio differs among sites. These results also indicated that the 13-mt genes of cephalopods are globally evolving under negative constraints, with a few percentage evolving under neutrality. However, since these models are not able to report which codons are under negative selection, we also performed FEL site-by-site analyses (p-value < 0.05). According with these analyses, the three mt subunits (COX1, COX2 and COX3) belonging to Complex IV presented the highest percentage of codons under negative selection–with COX1 subunit standing out with 89% of codons under negative selection (Fig 2A)–as well as, the greatest efficient purifying selection (smaller ϖ value) (Fig 2B). CYTB from Complex III also revealed a high value of purifying selection contrasting with the subunits from Complexes I and V, which showed more relaxed selection (Fig 2B). In particular, the subunits having the highest percentage of codons under negative selection present the following order: COX1 > COX3 > COX2 > ATP6 > ND1 > CYTB > ND3 > ND5 > ND4 > ND6 > ND2 > ND4L > ATP8 (Fig 2A). Additionally, it was possible to verify that the subunits under stronger purifying selection (smaller ϖ) display the following order: COX1 > COX2 > CYTB > COX3 > ND1 > ATP6 > ND3 > ND5 > ND4L > ND2 > ND4 > ND6 > ATP8 (Fig 2B). Both analyses confirmed, similarly to vertebrate species, the great influence of purifying selection in the 13-mt subunits of our Cephalopoda dataset (Fig 2 and S8 Table).

### Evidence of natural selection in Cephalopoda mitochondrial genes

**Fig 3:**
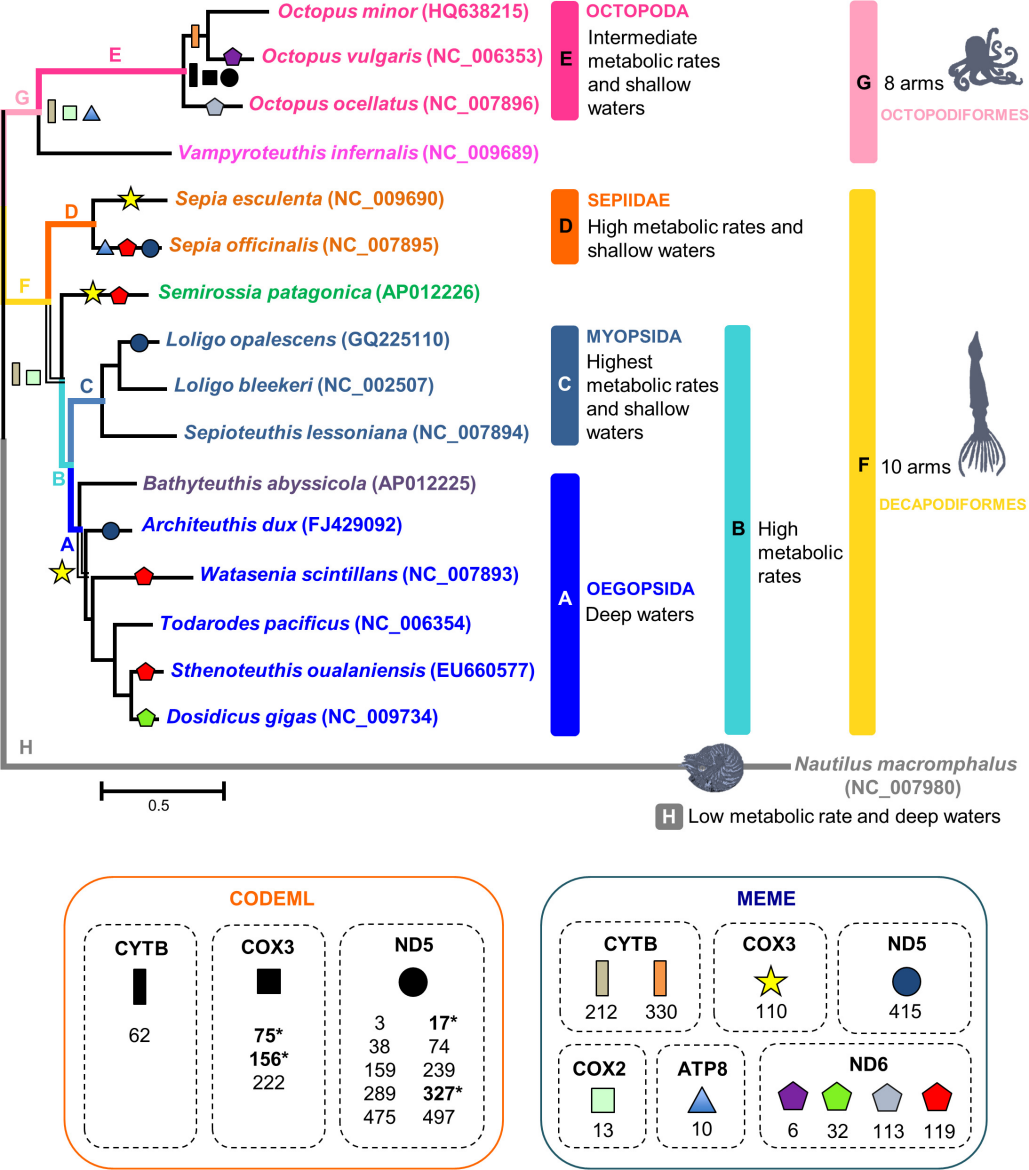
Foreground-branches tested for both branch-specific and branch-site selection models. Identification of the Maximum likelihood tree branches to test the adaptive evolution for each one of the 13-mitochondrial genes in 17 representative species from the molluscan Class Cephalopoda. The letters indicate the branches of interest (foreground-branches: named A to H). We performed 8 tests, where only one of the branches pointed by the letters was considered at a time; all other branches are corresponding to background-lineages for the analyzed gene. The sites represented correspond to positively selected sites by the employed approaches (CODEML and MEME). The numbers in bold, with an asterisk mark, represent sites that obtained posterior probabilities ≥ 99% (p-value < 0.05) and the other numbers, posterior probabilities ≥ 95% (p-value < 0.05), respectively.

From the employed branch (CODEML) and branch-site specific (CODEML from PAML and MEME from DATAMONKEY web-server) analyses, considering several branches of interest (foreground-branches), only the branch-site specific analyses (p-value < 0.05) uncovered events of positive selection along the ML mitochondrial phylogeny of cephalopods (Fig 3).

#### Branch analyses

For each one of the foreground-branches tested (A to H) (Fig 3), branch model analyses from CODEML (p-value < 0.05) revealed that globally (S9 Table): (A) the mt genes of species living in deep waters, compared with species inhabiting different depths, are evolving under negative selection, with the exception of *cox2* and *cox3* which appeared to be evolving under relaxed selective constraints; (B) the mt genes of squid species (with high metabolic rates) when compared with the remaining groups (distinct metabolic rates and depths) are also evolving under negative selection, with the exception of the *cox2*, *cox3*, *cytb* and *nd1*, evolving under relaxed selective constraints; (C) the Loliginidae family, with the highest metabolic rate, presents the *cox1*, *cox2*, *cox3* and *nd5* genes evolving under relaxed selective constraints and the remaining nine genes, evolving under negative selection, at the same rate, in all the lineages of cephalopods; (D) in Sepiidae group (high metabolic rate and species living in shallow waters) all the mt genes are evolving under negative selection, at the same rate of the other cephalopods, with the exception of the *cox1*, *cox2*, *cox3*, *cytb* and *nd5* genes that are evolving under relaxed selective constraints; (E) in Octopoda species (intermediate metabolic rates and shallow waters) all the genes are under negative selection (similar rates to the other species), except the *cox1* that is evolving neutrally; (F) in Decapodiformes all the genes are evolving under negative selection (the same rate of Octopodiformes), except for *cox1* and *cox3* evolving neutrally; (G) in Octopodiformes all the genes are evolving under negative selection, except the *cox1* that is evolving neutrally; (H) in the *Nautilus macromphalus* species, all the mt genes are evolving under negative selection, at the same rate of all the other cephalopods (branches), with the exception of the genes *cox1* and *cytb* which have evolutionary rates (ϖ*cox1* = 0.036 and ϖ*cytb* = 0.002) distinct from the other cephalopods.

#### Branch-site analyses

From the branch-site model tests of CODEML (S10 Table), the BEB analyses (posterior probabilities ≥ 95%) were able to identify a few codons under positive selection (p-value < 0.05) for the genes *nd5*, *cytb* and *cox3* in the ancestral lineage of the Octopoda group (foreground-branch E) (Fig 3 and S10 Table). Additionally, MEME analyses (posterior probabilities ≥ 95%) also reported sites with evidence of episodic diversifying selection (p-value < 0.05) in the following mt subunits: *nd5*, *nd6*, *cytb*, *cox2*, *cox3* and *atp8* (Fig 3). It is worth highlighting that MEME analyses revealed the presence of codons under positive selection in *cytb*, *cox2* and *atp8* genes in the ancestral lineage of Octopodiformes (Fig 3 -foreground-branch G). Assuming as reference the protein sequences of the *Octopus vulgaris*, the positively selected sites were the following (Fig 3): from CODEML (i) ND5 subunit—3, 17, 38, 74, 159, 239, 289, 327, 475 and 497, CYTB subunit—62 and COX3 subunit—75, 156 and 222; and from MEME (ii) ND5 subunit—415, ND6 subunit—6, 32, 113 and 119, CYTB subunit—212 and 330, COX2 subunit—13, COX3 subunit—110 and ATP8 subunit—10 (Fig 3).

### Mapping of the positively selected and other functional relevant sites

The implemented methodological approaches revealed sites under positive selection in only six of the 13 protein-coding genes considered in this study: *nd5*, *nd6*, *cytb*, *cox2*, *cox3* and *atp8* (p-value < 0.05).

#### *ND5* subunit

**Fig 4:**
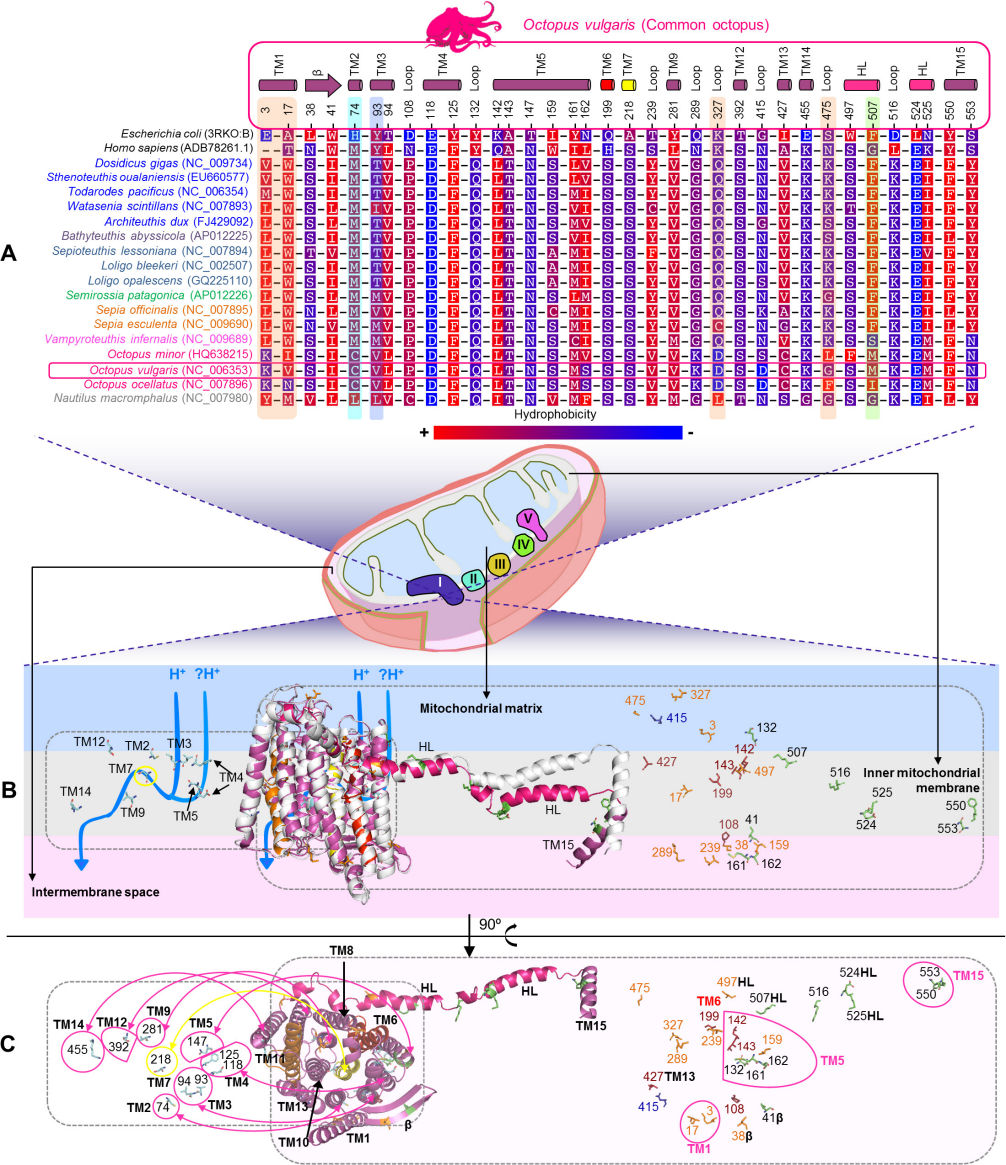
Amino acid variation in functional and structural sites of Cephalopoda ND5 subunit. (A) Amino acid alignment for sites (i) under positive selection—3, 17, 38, 74, 159, 239, 289, 327, 475, 497 (CODEML branch-site model test) and 415 (MEME), (ii) sites involved in interactions between subunits—41, 132, 161, 162, 507, 516, 524, 525, 550, 553 (iii) sites lining proton translocation channels—74, 93, 94, 118, 125, 147, 218, 281, 392, 455 and (i) described mutations—108, 142, 143, 199 and 427. All the sites have as reference number, the site position from the *Octopus vulgaris* ND5 protein-sequence, highlighted with a magenta rectangle shape. Sites highlighted by: (i) an orange shape—identified by CODEML branch-site model test and employing TREESAAP analysis; (ii) cyan shape—site lining proton translocation channel and reported by CODEML branch-site model test; (iii) blue shape—site lining proton translocation channel and positively selected by TREESAAP; (iv) green shape—site involved in subunit interactions (ND5-ND4) and also positively selected by TREESAAP. Background amino acid colors represent a hydrophobicity scheme from the most hydrophobic (red) to the most hydrophilic (blue). (B) Superimposition of 3D structures of ND5 from *Octopus vulgaris* (magenta) and NuoL from *Escherichia coli* (white), viewed from inner mitochondrial membrane—map of all the previously mentioned sites. At the left are projected the sites (cyan sticks) lining the proton translocation channels (blue arrow): H^+^—putative channel and? H^+^—suggested channel at the interfaces of ND5 and ND4 subunits. At the right, the projected sites are involved in interactions between subunits (green sticks), described mutations (red sticks) and the sites under positive selection (CODEML—orange sticks; MEME—blue sticks). (C) View from the intermembrane space. TM—transmembrane helices: helices that are probably involved in conformational changes are shown in red (TM6—discontinuous helix), orange (TM11—discontinuous helix) and yellow (TM7—discontinuous helix). Connecting elements: β—β-hairpins and HL—HL helix (hot pink).

The sites identified as positively selected by branch-site analyses (p-value < 0.05) were mapped in the Cephalopoda ND5 protein sequence alignment (Cephalopoda ND5 dataset) (Fig 4A) and into the predicted *Octopus vulgaris* 3D protein-structure (Fig 4B and 4C), allowing to localize the sites 3, 17, 74, 159 and 497 (CODEML) in TM helices, the site 38 in a β sheet (CODEML) and the sites 239, 289, 327, 475 (CODEML) and 415 (MEME) in loop regions (Fig 4). The profile alignment of the Cephalopoda ND5 dataset with the structure-based alignment from a recent study (using 30 representatives from all kingdoms of life: vertebrates, plants, algae, bacteria, yeasts, fungi and cnidarians) [68] (S3 Table), indicated that in cephalopods this protein has 15 TM helices. This was also illustrated by the superimposition of the predicted *Octopus vulgaris* 3D protein-structure with the X-ray 3D structure of the NuoL (a counterpart of the ND5), from the bacterium *Escherichia coli* (*E*. *coli*, PDB code 3RKO), which revealed that the TM missing in cephalopods corresponds to the TM1 of the bacterium (Fig 4). Among the mentioned sites, only the 74 and the 497 presented a substantial level of conservation (Fig 4A) in the Cephalopoda ND5 dataset. Noteworthy that the Octopoda species have a positively selected cysteine (C) amino acid in the position 74 of TM2, instead of a methionine (M) amino acid (M74C), which is present in the remaining cephalopods, with exception of the *Nautilus macromphalus* (Fig 4A). Thus, Octopoda species have a polar uncharged amino acid, soluble in water, instead of a non-polar hydrophobic one, present in the other cephalopods. Our homology comparisons (Fig 4A) suggested that this site–homologous of the site H100 in *E*. *coli* [68] (S3 Table)–is part of the proton translocation channel, across the inner mitochondrial membrane to enable ATP production, being one of the three amino acids that form its main link to the mitochondrial matrix in cephalopods (Fig 4B). The following sites– 3 (TM1), 159 (TM5) and 239 (loop)–presented average amino acid conservation (Fig 4A). Additionally, TREESAAP analysis (p-value < 0.001) also reported that the site 3 presents the amino acid property polarity under positive selection. The sites 17 (TM1) and 475 (loop) showed more variable amino acid changes (Fig 4A). Accordingly, TREESAAP analysis (p-value < 0.001) revealed the following amino acid properties under positive selection: polarity at the codon 17 and chromatographic index, hydropathy and surrounding hydrophobicity at the codon 475. The codon 17 is variable (Fig 4A and S3 Table) among the 30 representative species of all kingdoms of life [68], with contrasting hydrophobicity and polarity. In our Cephalopoda ND5 dataset the hydrophobicity of the amino acids in the referred position is well conserved (exception *Octopus ocellatus*) (Fig 4A). Furthermore, the tryptophan (W17) amino acid is present in all Decapodiformes, which could be suggestive of an important adaptive role in the referred group (Fig 4A). The remaining positively selected sites, 38 (β sheet), 289 (loop) and 327 (loop), present average conservation (Fig 4A) and the TREESAAP analysis (p-value < 0.001) was able to identify the polarity property as positively selected at the codon 327. The amino acid present in the site 415 (loop) is highly variable, being mostly hydrophilic (Fig 4).

Additionally, through homology comparisons based on the sites described by Efremov & Sazanov (2011) (for *E*. *coli* and/or *Homo sapiens*) [68], which are involved in interactions between subunits, mutations and residues lining proton translocation channels, we were able to determine the sites that present different amino acids in homolog positions of cephalopods (Fig 4 and S3 Table). It is worth highlighting two of these sites, 93 and 507, to which the TREESAAP (p-value < 0.001) revealed amino acid properties under positive selection. The codon 93 (homologous to Y119 of *E*. *coli*) is referred as a possible link of the putative proton translocation channel at subunits interface [68] (Fig 4A and S3 Table). This suggested function could be maintained in Oegopsida (with exception of *Watasenia scintillans* species), Bathyteuthoidea and Myopsida, since at this position these species have a polar threonine (T93) amino acid that may participate in hydrogen bonds (Fig 4A). However, in the case of the remaining cephalopods that have non-polar amino acids (M, V-valine and L-leucine) (Fig 4A), incapable of forming hydrogen bonds, this interface link could be compromised. The TREESAAP analysis (p-value < 0.001) revealed, for this site, the following properties under positive selection: solvent accessible reduction ratio and surrounding hydrophobicity; being in accordance with the previously described amino acid substitutions. The codon 507 is located in a structural element (Fig 4—HL helix)–which extends along nearly the entire length of the hydrophobic membrane-embedded domain of Complex I, composed by the seven NADH dehydrogenase mt subunits–that may act as a connecting element (S2 Fig), coordinating conformational changes. For this site, the TREESAAP analysis (p-value < 0.001) indicated the amino acid properties bulkiness, solvent accessible reduction ratio and surrounding hydrophobicity, as positively selected.

#### *ND6* subunit

**Fig 5:**
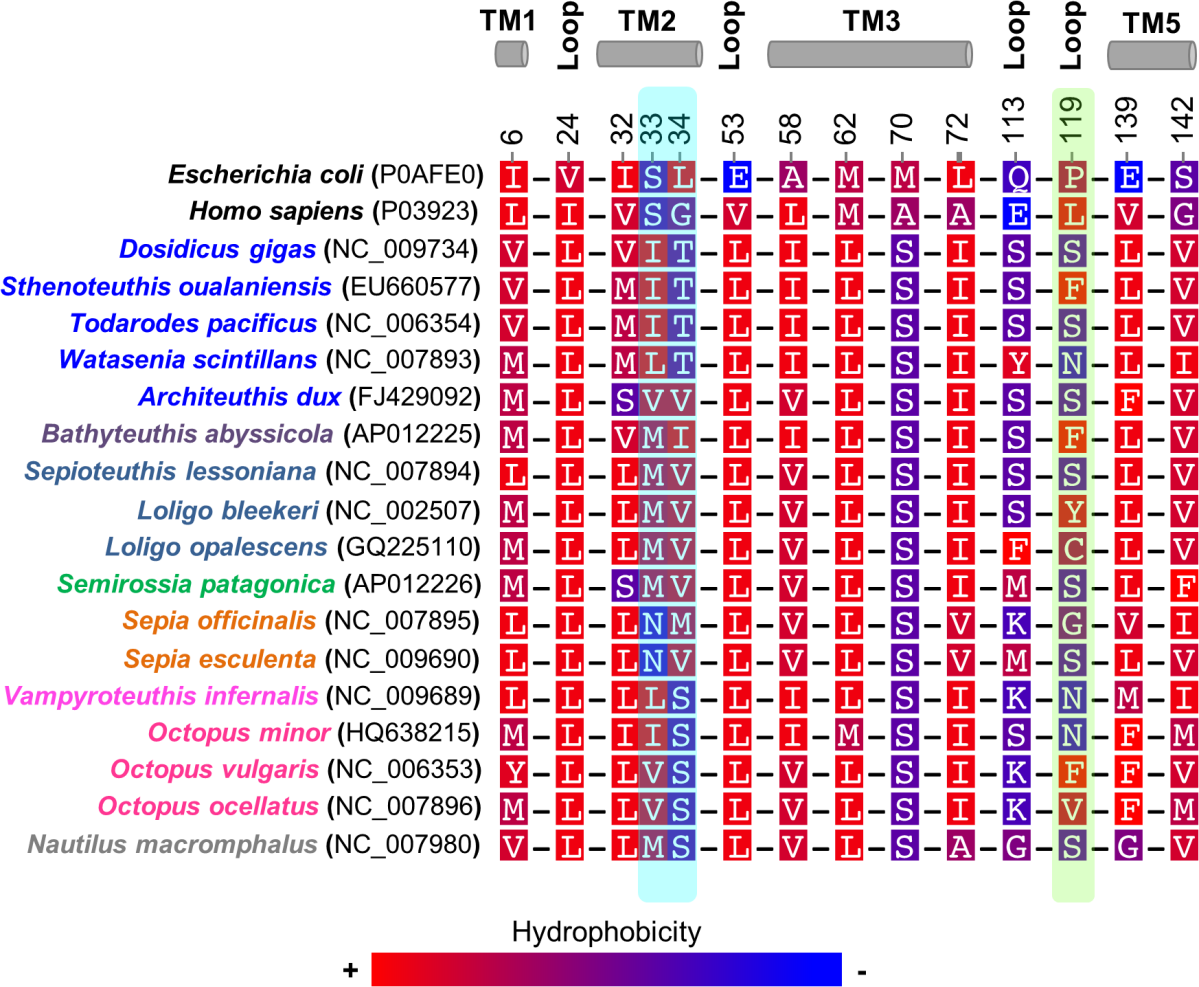
Amino acid variation in functional and structural sites of Cephalopoda ND6 subunit. Amino acid alignment for sites: (i) under positive selection—6, 32, 113 and 119 (MEME); (ii) at the surface—24 and 72; (iii) lining proton translocation channels—33, 34, 53, 58, 62, 70, 139 and 142. All the sites have as reference number the site position from the *Octopus vulgaris* ND6 protein-sequence. The sites lining proton translocation channel also identified by TREESAAP are highlighted with a cyan shape and with a green shape the sites positively selected by MEME and also by TREESAAP. Background amino acid colors represent a hydrophobicity scheme from red—the most hydrophobic to blue—the most hydrophilic. TM—transmembrane helices. TM and loops—according to the topology TMHMM prediction.

For the ND6 subunit was not possible to acquire a satisfactory 3D model. Therefore, insight into the evolution of this mt subunit in cephalopods was obtained by inspection of the amino acid substitutions, positively selected by MEME (p-value < 0.05) and TREESAAP (p-value < 0.001), observed in the sequence alignment of the cephalopods (Fig 5). Furthermore, sites relevant for the proton translocation and stability of the complex [68] were also considered (Fig 5 and S4 Table).

Employing the MEME analysis (p-value < 0.05) was possible to detect the following sites– 6 (TM1), 32 (TM2), 113 (loop) and 119 (loop)–evolving under positive selection in Cephalopoda species. The site 6 presents amino acids varying between V, M, L and tyrosine (Y) (Fig 5). All of these amino acids have hydrophobic side chains, however only the Y amino acid can form hydrogen bonds. The profile alignment of the Cephalopoda ND6 dataset with the structure-based alignment containing the NuoJ (*E*. *coli*: counterpart of Cephalopoda ND6) [68] (S4 Table) suggests that the site 32 is adjacent to amino acids close to a fourth proton translocation channel (Fig 5—cyan shape) at the interface of subunits ND2, ND4L, ND6 and ND3 [68]. At this site, the most drastic amino acid change occurs in the *Architeuthis dux* and *Semirossia patagonica*, both with a serine (S) amino acid. This is a small hydrophilic amino acid capable of forming hydrogen bonds, contrasting with the V, M, L and isoleucine (I) of the remaining cephalopods (Fig 5). Observing the Fig 5 it is possible to see that the site 113 (loop), is mainly dominated by hydrophilic amino acids with the exception of four species of Decapodiformes, that have a Y, phenylalanine (F) or M in this position. Considering the predicted topology (S3 Fig), this site is facing the mitochondrial matrix. The site 119 seems to be a hyper variable site (Fig 5—green shape), comprising amino acids with distinct properties and sizes (cephalopods: S, F, asparagine (N), Y, C or glycine (G)). Accordingly, TREESAAP analysis (p-value < 0.001) also revealed the following amino acid properties as being under strong positive selection: hydropathy, solvent accessible reduction ratio, polarity and chromatographic index.

Furthermore, our analysis suggested that the site L24, conserved in all the cephalopods (Fig 5), is a counterpart of the site 26 of *Homo sapiens* (S4 Table), for which a described mutation I26M was associated with [67]: loss of vision, caused by the Leber Hereditary Optic Neuropathy (LHON) disease and severe Complex I deficiency. Its homolog site in *E*. *coli* (V26) (Fig 5 and S4 Table) is located at the surface and is suggested to be near the probable quinone binding site (Q-site), formed at the interface of subunits NuoH (ND1), NuoJ (ND6) and NuoA (ND3) with the hydrophilic peripheral domain of Complex I [84]. We also found that the sites 33 and 34 (Fig 5—cyan shape), homologs of S35 and L36 of *E*. *coli* (S4 Table), can be lining a proton translocation channel [68]. These sites were also reported by TREESAAP, which indicated the following amino acid properties, as being under strong positive selection (p-value < 0.001): (i) site 33 –polarity and (ii) site 34 –surrounding hydrophobicity and solvent accessible reduction ratio. Additionally, it was described the mutation G36S in *Homo sapiens*, which occurs in a site homolog of the codon 34 in cephalopods (S4 Table). This mutation was associated with the LHON disease [82] and consists in the replacement of a non-polar residue (G) by an amino acid with a polar uncharged side chain (S).

#### *CYTB* subunit

**Fig 6:**
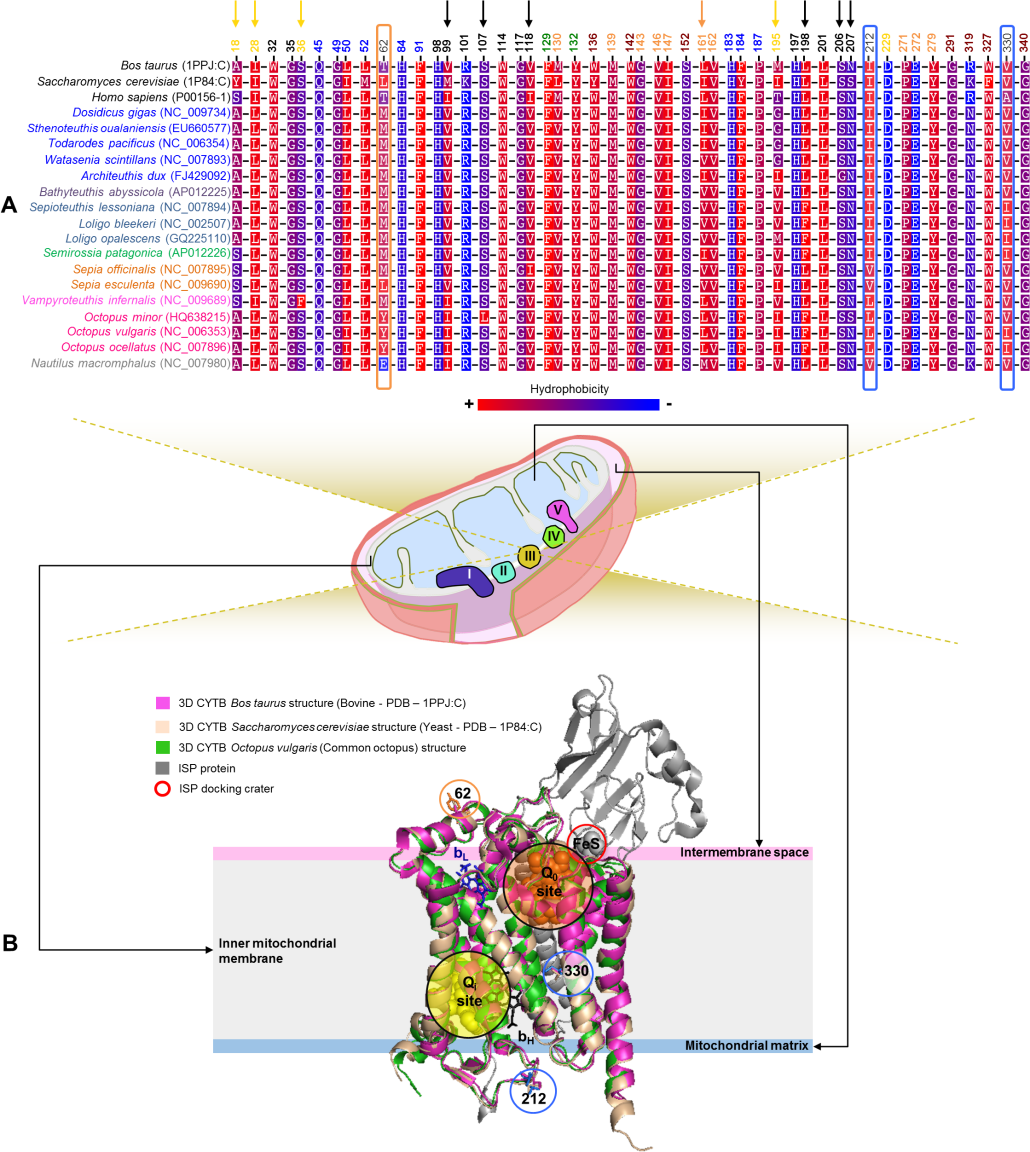
Amino acid variation in functional and structural sites of Cephalopoda CYTB subunit. (A) Amino acid alignment for sites (i) under positive selection—orange shape (CODEML branch-site model test) and blue shape (MEME), (ii) mutations related with human exercise intolerance—maroon sites, (iii) Q_o_ site binding sites—orange sites, (iv) Q_i_ site binding sites—yellow sites, (v) b_L_ binding sites—blue sites and (vi) b_H_ binding sites—black sites. The amino acid substitutions in some of the mentioned sites are marked with arrows. (B) Superimposition of 3D structures with the positively selected sites mapped as sticks (orange for CODEML and blue for MEME). All the sites have as reference number, the site position from the *Octopus vulgaris*.

The positively selected sites found by CODEML branch-site model test (62) and MEME analyses (212 and 330) (p-value < 0.05), were mapped in the corresponding alignment (Fig 6A) and into the predicted 3D protein-structure (Fig 6B). The mapping of these sites allowed to verify that the site 62 is well conserved in cephalopods, except for the *Sepia esculenta*, Octopoda species and the *Nautilus macromphalus* (Fig 6A—orange shape). Overall, despite the amino acid replacements, the hydrophobicity of this site is maintained. The exception is the Nautiloidea which presents in this site an amino acid, glutamic acid (E), highly hydrophilic. Observing the CYTB 3D protein-structure (Fig 6B—orange shape) it was also possible infer the location of this site in a helix named *ab*, outside the membrane and parallel to the membrane plane. This is an amphipathic helix and thus the amino acid replacements verified do not cause detrimental structural changes, being possibly advantageous for these species. Both sites 212 and 330 have amino acid substitutions conserved in subgroups of cephalopods, with distinct metabolic rates, all keeping the hydrophobicity of the sites (Fig 6A—blue shapes). The 3D protein-structure revealed that the site 212 is located in a loop region facing the mitochondrial matrix and the site 330 is located in the transmembrane helix G (Fig 6B—blue shapes). Furthermore we mapped, in the corresponding alignment (Fig 6A) relevant functional binding sites, obtained through homology comparisons with well-studied CYTB X-ray crystal structures from *Bos taurus* [70, 83] and *Saccharomyces cerevisiae* [81], as well as, mutations related with *Homo sapiens* exercise intolerance [75]. These analyses demonstrated the high degree of conservation among CYTB sequences in cephalopods (Fig 6A), even at sites for which have been reported exercise intolerance associated mutations in humans [75] (Fig 6A—mapped in maroon). Examples of extreme conservation are the following relevant sites, inferred in cephalopods: F129, Y132, E272 and Y279 (Fig 6A and S5 Table). The site 129 presumably stabilizes the hydrophobic tail of the ubiquinol, proposed to center quinol oxidation (Q_o_) pocket [81]. The hydroxyl group of the tyrosine at the site 132, located between E272 and the prosthetic group low-potential *b* heme (b_L_), is thought to stabilize the proton transfer pathway [81]. The site 272, located in the transition between the transmembrane F1 and a loop region, is proposed to be a ligand for ubiquinol and to accept protons released during ubiquinol oxidation [81]. The residue 279 is proposed to pre-orient the substrate [81].

Accordingly, TREESAAP did not showed properties under positive selection (p-value < 0.001) for any of these sites. However, by inspection of other relevant sites in the sequence alignment, which are forming the Q_o_ site, ubiquinone reduction (Q_i_) site and the b_L_ binding sites [70, 83], we found amino acid substitutions in some of the mentioned sites (Fig 6A—marked with arrows). The *Vampyroteuthis infernalis* has two atypical amino acid replacements at positions 28 and 36 (Fig 6A—yellow arrows) that are conserved in all the other members of Class Cephalopoda. Both residues are located at the Q_i_ site (Fig 6 and S5 Table), suggesting that these amino acid replacements will affect the binding of ligands at this site. Furthermore, the sites 18 and 195 (both from Q_i_ site) evidenced amino acid changes conserved in subgroups of cephalopods, where the hydrophobicity is changed (Fig 6A—yellow arrows). The site 161 belonging to the Q_o_ site (S5 Table), has a L conserved in all the Octopodiformes, alternating between an I/V in the other cephalopods, or M in *Nautilus macromphalus* (Fig 6A—orange arrow). The *Octopus minor* species presents an atypical amino acid replacement, in cephalopods, in the sites 107 and 207 (Fig 6A—black arrows), both belonging to the prosthetic group high-potential *b* heme (b_H_) binding site (S5 Table). Similarly, the *Architeuthis dux* is the only species having a G instead of S at the site 206 (b_H_ binding site) (Fig 6A—black arrow). The sites 99 and 198 (Fig 6A—black arrows), both b_H_ binding sites, present amino acid substitutions keeping the hydrophobicity of the sites.

#### *COX2* subunit

**Fig 7:**
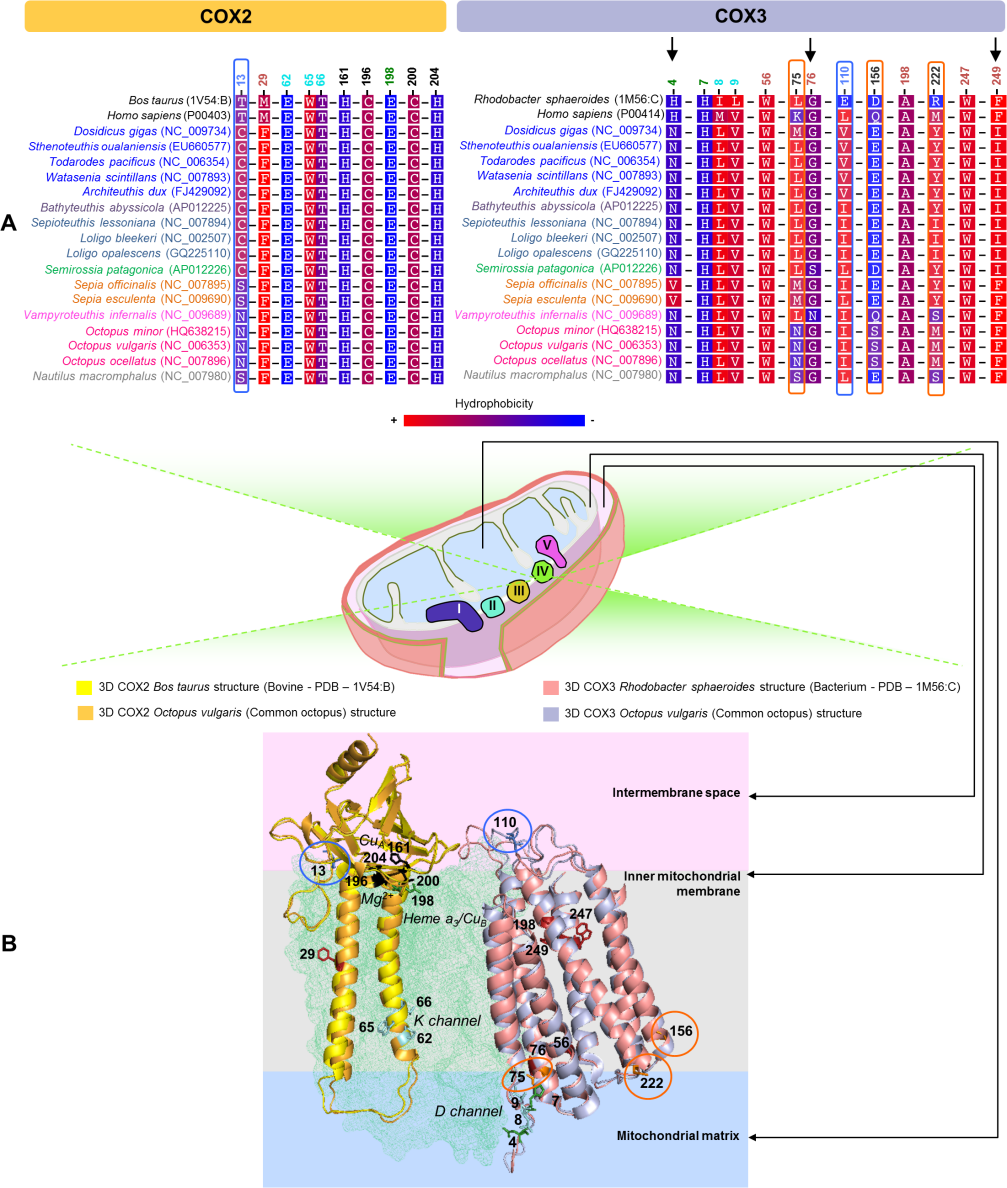
Amino acid variation in functional and structural sites of Cephalopoda COX2 and COX3 subunits. (A) COX2: Amino acid alignment for (i) sites under positive selection—blue shape (MEME), (ii) mutations related with human exercise intolerance—red sites, (iii) Cu_A_ metal binding sites—black sites, (iv) sites involved in proton coupling mechanism—cyan sites and (v) metal binding site for Mg^2+^—green site. COX3: Amino acid alignment for (i) sites under positive selection—orange shape (CODEML branch-site model test) and blue shape (MEME), (ii) sites in the proximity of the entrance of D-channel—green sites, (iii) residues that protect the entrance of the D-channel from direct solvent exposure—cyan sites and (iv) sites related with diseases in humans—red sites. The amino acid substitutions in some of the mentioned sites are marked with arrows. (B) Superimposition of 3D structures with the sites mapped as sticks (colors according to Fig 7A). All the sites have as reference number, the site position from the *Octopus vulgaris*.

From the analyses performed only the site 13 presented evidences to be evolving under positive selection, according to MEME (p-value < 0.05) and to TREESAAP (p-value < 0.001) results. The inspection of the Cephalopoda COX2 protein sequence alignment revealed the presence of a conserved amino acid (C) in Oegopsida, Bathyteuthoidea, Myopsida and Sepiolidae groups at this site (Fig 7A). At the same position the other cephalopods have an amino acid, with polar uncharged side chain, S or N (Fig 7A). Accordingly, for this site, TREESAAP showed the amino acid property polarity under positive selection (p-value < 0.001). The mapping of this site into the predicted 3D protein-structure revealed that it is located in a loop region facing the intermembrane space (Fig 7B).

The homology comparisons performed (S6 Table) showed that the well conserved residues E62, W65 and T66 of cephalopods (Fig 7—cyan sites) correspond to residues of particular interest in relation to proton coupling mechanism, since they form hydrophilic channels that provide the necessary routes for intra protein proton movements [73]. These analyses also pointed that the Cu_A_ metal binding sites H161, C196, C200 and H204 (S6 Table) are conserved in all the cephalopods (Fig 7—black sites), highlighting its functional importance. Furthermore, we mapped the site 29, to which was reported a mutation (M29K) that causes exercise intolerance in humans [79] (S6 Table), showing a conserved amino acid (F) in all the cephalopods (Fig 7A). We also mapped the E198, metal binding site for Mg^2+^ [73] (S6 Table), which is equally conserved in all the cephalopods.

#### *COX3* subunit

The mapping of the positively selected sites (p-value < 0.05) into the predicted 3D protein-structure showed that the sites 75 and 156 are located in transmembrane helices and the sites 110 (intermembrane space) and 222 (mitochondrial matrix) in loop regions, respectively (Fig 7B). TREESAAP indicated as positively selected (p-value < 0.001) the following amino acid properties: polarity for the site 75 and solvent accessible reduction ratio for the site 222. Through homology comparisons (S7 Table), we observed that the site 75 is adjacent to a site (76) to which a disease related mutation (G78S—LHON) was described in humans (Fig 7) [73]. The considerable polarity change, observed in the site 75 of the species *Octopus minor* (L75N), *Octopus vulgaris* (L75N), *Octopus ocellatus* (L75N) and *Nautilus macromphalus* (L75S) (Fig 7A), was also reported by TREESAAP (p-value < 0.001). These sites could be important for the stabilization of the Complex IV thereby enhancing ATP production proper to each species with different habitats and metabolic requirements. We also mapped other relevant sites, such as L8 and V9 (S7 Table), conserved in all the cephalopods. These hydrophobic residues protect the entrance of the D-channel in COX1 from direct solvent exposure [63]. The homology analyses (S7 Table) revealed that the sites 4 and 7 in cephalopods are counterparts of the sites H7 and H10 of the *Rhodobacter sphaeroides*, respectively. These sites are also in the proximity of the entrance of D-channel. A recent study indicated that mutations in these sites (H7Q and H10Q) indirectly lower the activity of the complex, by slowing the uptake of protons through the D-channel, and that their histidine residues stabilize the interactions between COX1 and COX3 [63]. The inspection of the Cephalopoda COX3 protein sequence alignment showed that the histidine of the site 7 is conserved in all the cephalopods (Fig 7A). However, the site 4 presents an asparagine (N4) conserved in all the cephalopods with exception of the Sepiidae species that instead possess a valine (V4) (Fig 7A). Furthermore, TREESAAP reported the amino acid properties hydropathy and solvent accessible reduction ratio for the site 4. Therefore, these substitutions may have impact at the stabilization of the complex. From the sites corresponding to sites related with diseases in humans (S7 Table) we observed that they are mostly conserved in cephalopod species (Fig 7A—red sites). The exceptions occur at sites 76 and 249, both located in transmembrane helices (Fig 7B). At the site 76 it is possible to observe a mutation G76S in *Semirossia patagonica* and G76N in the *Vampyroteuthis infernalis* (Fig 7A). At the site 249 a mutation F249I occurs in the groups Oegopsida, Bathyteuthoidea, Myopsida and Sepiolidae (Fig 7A).

#### *ATP8* subunit

For the ATP8 subunit it was not possible to obtain a satisfactory 3D model. However, a topological assignment (S4 Fig) showed that the positively selected site 10 (MEME method, p-value < 0.05) is located in the transition of a loop, located in the intermembrane space, to a transmembrane helix. This site presents the hydrophobic residues L10 and I10; the former in the Oegopsida, Bathyteuthoidea, Myopsida, *Semirossia patagonica*, *Sepia esculenta* and *Nautilus macromphalus*, and the latter in the remaining cephalopods, respectively.

## Discussion

Overall, all the employed approaches for phylogenetic analyses–considering the concatenation of 13-mt genes of 17 representative species from the Cephalopoda major taxonomic groups–generated a robust mitochondrial phylogeny for the Class Cephalopoda. Additional support can be inferred for the few cases where only a moderate support was obtained for the species relationship (*e*.*g*. *Watasenia scintillans* and *Architeuthis dux* intra-relationships within the Oegopsida clade), through the observation that all the achieved tree topologies where equal. Furthermore, the mitochondrial topology (Fig 1) globally agrees with a recent phylogeny of 188 cephalopod species (which used mitochondrial and nuclear genes) [35]. The clades defined in the mitochondrial phylogeny are also corroborated when some biological and ecological characteristics are considered (number of arms, metabolic rates and depths) (Fig 3). In fact, we can observe that the Octopodiformes (8 arms) and Decapodiformes (10 arms) form the two major clades. Within these major clades the species grouped into several sub clades, which have cephalopods with identical metabolic rates and inhabiting similar ocean depths (Fig 3). Consequently, we expected that similar selective pressures could be shaping the evolution of mitochondrial genes of species within the same clade.

Our site model results (p-value < 0.05, S8 Table) indicated that the 13-mt genes of cephalopods are globally evolving under negative constraints (purifying selection), with a small percentage of codons evolving under neutrality, reinforcing their crucial and conserved role for the body energy production in cephalopods. This is also in agreement with the general trend of the mt genome evolution in vertebrates [85].

Given the high percentage of sites in the mt genes evolving under negative selection, evidences of pervasive positive selection (affecting few sites in particular linages) may be masked by the majority sign of negative selection in the branch-specific analyses. This is because these types of analyses are more conservative than branch-site model tests, thus justifying their distinct results.

From the several foreground-branches (A-H)–considering groups of cephalopods with different metabolic rates, depths and number of arms–branch-site model tests revealed sites under positive selection in particular lineages (Fig 3). It is worth highlighting the presence of a few codons under positive selection (p-value < 0.05) in the genes *nd5*, *cytb* and *cox3* (CODEML) in the ancestral lineage of the Octopoda group (foreground-branch E) (Fig 3 and S10 Table), and also in *cytb*, *cox2* and *atp8* genes (MEME) in the ancestral lineage of Octopodiformes (foreground-branch G) (Fig 3).

We have placed special attention to the codons with the hydrophobicity property under positive selection, since OXPHOS complexes are intricately linked to the mitochondrial inner membrane [86]. Thus, we would expect this property to be under particularly high purifying selection. Indeed, previous evidence covering four phylogenetic distant animal clades–mammals, birds, insects and nematodes–revealed that the negative ω value per clade was on average 3.9 times lower in mitochondrial OXPHOS genes (mt OXPHOS) than in nuclear OXPHOS genes, even restricting the mitochondrial-nuclear comparison to the same OXPHOS pathway [86]. This study refers with statistical support, that genes with high levels of hydrophobicity and expression, are those under the strongest selective constraint [86]. Moreover, as in the case of mt OXPHOS subunits, other studies revealed that highly expressed proteins have particularly detrimental effects when they are hydrophobic at the same time [87, 88]. Hence, this property can be important in protein-protein (mitochondrial-mitochondrial and mitochondrial-nuclear) interactions, reinforcing the role of these subunits in the OXPHOS pathway [86]. Additionally, a change in this functional property can compromise the folding of a subunit, or even interfere in the stability of the entire complex. If this situation occurs, the non-polar residues could invade other subunits and membrane areas causing dysfunctional protein-protein interactions and the disruption of membranes [86]. The inverse case is also possible, the occurrence of some variations that could support a more efficient process, conferring adaptive advantage.

Our results suggest that positive selection may have had particular effect on the subunits ND5 and ND6 of cephalopod species. This can be striking in the ND5 subunit, which is a highly hydrophobic protein, conserved from prokaryotes to eukaryotes, and critical for transducing conformational energy to proton-pumping elements in the distal module of the membrane arm of the Complex I [68]. According to a recent study, the lack of the TM1 helix in ND5 only occurs in arthropods and nematodes [68]. Our study also provides evidence of the lack of the TM1 helix in cephalopods. Furthermore, we also revealed for the first time that the positively selected site 74 is also part of the proton translocation channel in cephalopods. This relevant site was positively selected in the Octopoda lineage (foreground-branch E, p-value < 0.05), suggesting an adaptive advantage to these species with intermediate metabolic rates and inhabiting shallow waters. Regarding the ND6 subunit in cephalopods, the amino acid at the site 24 of this subunit–counterpart of the site 26 of *Homo sapiens*, associated with loss of vision [67]–is highly conserved. Therefore, we suggest that the existence of a L instead of a I/V (reported in *Homo sapiens* and *E*. *coli*, respectively) should have no negative impact in the vision of Cephalopoda species, because hydrophobicity is maintained at the mutated site in cephalopods (Fig 5). However, further studies are required to understand if the activity of the complex is changed, since this site is near the Q-site of Complex I. Furthermore, we observed the presence of distinct amino acid substitutions occurring in the sites 33 and 34 –suggested to be lining the proton translocation channel [68] (S4 Table)–that although changing the hydrophobicity of these sites, are conserved in subgroups of cephalopods. Through homology studies (S4 Table), we verified that a mutation (G36S) in the human counterpart of the cephalopod site 34 was associated with the LHON disease [82]. Similarly, in cephalopods we observed (Fig 5) that all the Octopodiformes and the Nautiloidea have a S in their counterpart site (34S). Furthermore, in the same position, most of the cephalopods living in deep waters and without cornea covering the eyes–*Dosidicus gigas*, *Sthenoteuthis oualaniensis*, *Todarodes pacificus* and *Watasenia scintillans*–have also an amino acid with a polar uncharged side chain (34T). On the other hand, species with cornea–Myopsida, Sepiolidae and Sepiidae (Fig 5)–have an amino acid with a hydrophobic non-polar side chain (V or M). Thus, the presence of a polar uncharged amino acid (34T or 34S) may have some impact in the vision of the referred species. Consequently, this can have a critical effect in Octopodiformes species since they have cornea and live in shallow waters, where predators abound.

Overall, our results showed that most of the sites related with mutations described in humans, such as exercise intolerance, have conserved amino acids in all the cephalopods (Figs 6A and 7A). Similarly, the homology comparisons showed that residues of particular interest, as those involved in proton coupling mechanisms (Fig 7A), are mostly well conserved in all the cephalopods, highlighting its vital functional importance.

Since the beginning of this work, more mt genomes from cephalopods (NC_017746, NC_017749, NC_020348, NC_021146, NC_022466, NC_022467, NC_022468, NC_022693, NC_022959 and NC_023257) became available, however these are from the same major taxonomic groups represented in our dataset. Thus, although these new mt genomes were not included in our analyses, the concatenation of mt genes from fully sequenced mt genomes of 17 species still representative of the Class Cephalopoda, covering the eight taxonomic groups Oegopsida, Bathyteuthoidea, Myopsida, Sepiolidae, Sepiidae, Vampyromorpha, Octopoda and Nautiloidea, and provided a coherent Cephalopoda mitochondrial phylogenetic tree.

Furthermore, we were able to assess the adaptive evolution of cephalopod mt genomes, through the identification of negative and positive selection. We considered several foreground-branches, in order to better comprehend the adaptive evolution of cephalopod lineages, well adapted to contrasting environments, namely diverse depths, and consequently temperatures, pressures, light, food, etc. In particular, the mapping of the identified positively selected sites in the Cephalopoda ND5 dataset, along with homology analyses, allowed to conclude that some of these sites are: (i) lining proton translocation channels such as site 74TM2 (ND5—CODEML), and (ii) involved in interactions between subunits, namely site 507HL (ND5—TREESAAP). Additional relevant sites were identified through inspection of multiple sequence alignments of subunits from cephalopods with their counterparts (bacteria, yeast, bovine and/or human), and/or through its superimposition. Our study provides valuable insight into the adaptive evolution of the mt genome in cephalopods, and its implications for their molecular mechanism of OXPHOS.

## Supporting Information

S1 Fig Mitochondrial genes included in Cephalopoda datasets. At the top are the names of the mitochondrial genes. The arrow shapes indicate their orientation in the mitochondrial genomes. Each one of the duplicated genes included in our datasets are identified, based on their relative positions in the mitochondrial genomes, as left and right side duplicated genes, respectively.
(DOCX)

S2 Fig Architecture of the membrane domain of *Octopus vulgaris* Complex I. At the bottom it is possible to observe a mitochondria scheme with the subunits composing the five mitochondrial OXPHOS complexes, named in roman numerals. The mitochondrial matrix is represented in light blue, the inner mitochondrial membrane in grey and the intermembrane space in light pink. At the top, a detailed view from the membrane plane of the Complex I. This was obtained by superimposition of the predicted 3D structures (I-TASSER) of the *Octopus vulgaris* (Common octopus) subunits with the entire Complex I from *Escherichia coli* (PDB code 3RKO). Subunits are colored as follows: ND5—magenta, ND4—navy blue, ND2—yellow, ND6—green, ND3—light green, ND4L—purple and ND1—beige. The remaining subunits are from the PDB code 3RKO. Helices that are probably involved in conformational changes are shown in red, orange and yellow. Helix HL is shown in hot pink (horizontal).
(DOCX)

S3 Fig Topological assignment of ND6 subunit of Complex I. Yellow circles show the sites that present high number of radically changing properties under positive destabilizing selection, according to TREESAAP.
(DOCX)

S4 Fig Topological assignment of ATP8 subunit of Complex V. Yellow circles show the sites that present high number of radically changing properties under positive destabilizing selection, according to TREESAAP.
(DOCX)

S1 Table The Cephalopoda dataset in study. In this table are provided the Accession numbers of all the Cephalopoda species contemplated in this study, as well as, more details about their: taxonomy (species scientific name, common name, Cephalopoda major taxa and family), habitat (ocean zones, temperatures and corresponding average depths) and metabolic rates. UNK—indicates missing data.
(DOCX)

S2 Table Mutations in the duplicated mitochondrial genes detected in our Cephalopoda dataset. Green—highlights synonymous substitutions. Red—highlights non-synonymous substitutions. (=) means that both the genes are equal copies, without any nucleotide change. Nucleotides are abbreviated with the one-letter codes, as well as the amino acids. Example: *Dosidicus gigas*—considering the cox3 gene, A568G means that in the nucleotide number 568 an adenine (A) was changed by a guanine (G) nucleotide, resulting in a non-synonymous mutation in the codon number 190. At the amino acid level it is reflected in the change of an isoleucine (I) by a valine (V) amino acid. The substitution number position has as reference the gene that first appears in the mt genome (left side), comparing it with the second copy positioned after (right side duplicated gene). Oegopsida—without cornea. Myopsida—with cornea.
(DOCX)

S3 Table Homology analyses of the ND5 subunit. The sites identified as positively selected by branch-site analyses (CODEML and MEME: p-value < 0.05) were mapped in the Cephalopoda ND5 protein sequence alignment (Cephalopoda ND5 dataset: obtained through the translation of the respective MUSCLE codon based CDS alignment, performed in SEAVIEW software version 4.4.0). Then, we performed a profile alignment (using the GENEIOUS software version 5.6.7 *profile align* option) of the (i) Cephalopoda ND5 dataset (17 species) with the (ii) structure-based alignment (containing 30 representative species from all kingdoms of life) of the ND5 subunit from the study of Efremov & Sazanov (2011) [1]. Thus, we obtained a correspondence of the positively selected sites numbering (assuming as reference the ND5 protein sequence of the *Octopus vulgaris*) to the sequence numbers of species (*Escherichia coli* and *Homo sapiens*) with described residues involved in interactions between subunits, forming proton translocation channels and with associated mutations. TREESAAP is mentioned when a site also presented amino acid properties positively selected (p-value < 0.001).
(DOCX)

S4 Table Homology analyses of the ND6 subunit. The sites identified as positively selected by branch-site analyses (MEME: p-value < 0.05) were mapped in the Cephalopoda ND6 protein sequence alignment (Cephalopoda ND6 dataset: obtained through the translation of the respective MUSCLE codon based CDS alignment, performed in SEAVIEW software version 4.4.0). Then, we performed a profile alignment (using the GENEIOUS software version 5.6.7 *profile align* option) of the (i) Cephalopoda ND6 dataset (17 species) with the (ii) structure-based alignment (containing 30 representative species from all kingdoms of life) of the ND6 subunit from the study of Efremov & Sazanov (2011) [1]. Thus, we obtained a correspondence of the positively selected sites numbering (assuming as reference the ND6 protein sequence of the *Octopus vulgaris*) to the sequence numbers of species (*Escherichia coli* and *Homo sapiens*) with described residues involved in interactions between subunits, forming proton translocation channels and with associated mutations. TREESAAP is mentioned when a site also presented amino acid properties positively selected (p-value < 0.001).
(DOCX)

S5 Table Homology analyses of the CYTB subunit. The sites identified as positively selected by branch-site analyses (CODEML and MEME: p-value < 0.05) were mapped in the Cephalopoda CYTB protein sequence alignment (Cephalopoda CYTB dataset: obtained through the translation of the respective MUSCLE codon based CDS alignment, performed in SEAVIEW software version 4.4.0). (i) Then, we performed the superimposition (structure-based alignment) of the available CYTB X-ray crystal structures (*Bos taurus* PDB 1PPJ:C and *Saccharomyces cerevisiae* PDB 1P84:C) with the corresponding CYTB 3D structure of *Octopus vulgaris* (predicted in this study), using the PYMOL software version 1.5.0.4. Thus, we obtained a correspondence of the positively selected sites numbering (assuming as reference the CYTB protein sequence of the *Octopus vulgaris*) to the sequence numbers of species (*Bos taurus* and *Saccharomyces cerevisiae*), with relevant functional binding sites described. (ii) We also performed a MUSCLE alignment (in the SEAVIEW software version 4.4.0) of the Cephalopoda CYTB dataset with its homolog from *Homo sapiens*. This approach allowed to establish a correspondence of sites with described mutations causing diseases in humans, between *Homo sapiens* and cephalopods (*e*.*g*. *Octopus vulgaris*). Finally, we performed a profile alignment (using the GENEIOUS software version 5.6.7 *profile align* option) of the previous described alignments (i and ii), which allowed a correspondence of the sites among all the mentioned species.
(DOCX)

S6 Table Homology analyses of the COX2 subunit. The site identified as positively selected by branch-site analyses (MEME: p-value < 0.05) was mapped in the Cephalopoda COX2 protein sequence alignment (Cephalopoda COX2 dataset: obtained through the translation of the respective MUSCLE codon based CDS alignment; performed in SEAVIEW software version 4.4.0). (i) Then, we performed the superimposition (structure-based alignment) of the available COX2 X-ray crystal structure (*Bos taurus* PDB 1V54:B) with the corresponding COX2 3D structure of *Octopus vulgaris* (predicted in this study), using the PYMOL software version 1.5.0.4. Thus, we obtained a correspondence of the positively selected site numbering (assuming as reference the COX2 protein sequence of the *Octopus vulgaris*) to the COX2 sequence numbers of *Bos taurus* species, which has described sites involved in proton coupling mechanisms (K-channel) and functional binding sites. (ii) We also performed a MUSCLE alignment (in the SEAVIEW software version 4.4.0) of the Cephalopoda COX2 dataset with its homolog from *Homo sapiens*. This approach allowed to establish a correspondence of described sites mutations related with exercise intolerance in humans, between *Homo sapiens* and cephalopods (*e*.*g*. *Octopus vulgaris*). Finally, we performed a profile alignment (using the GENEIOUS software version 5.6.7 *profile align* option) of the previous described alignments (i and ii), which allowed a correspondence of the sites among all the mentioned species. TREESAAP is mentioned when a site also presented amino acid properties positively selected (p-value < 0.001).
(DOCX)

S7 Table Homology analyses of the COX3 subunit. The sites identified as positively selected by branch-site analyses (CODEML and MEME: p-value < 0.05) were mapped in the Cephalopoda COX3 protein sequence alignment (Cephalopoda COX3 dataset: obtained through the translation of the respective MUSCLE codon based CDS alignment; performed in SEAVIEW software version 4.4.0). (i) Then, we performed the superimposition (structure-based alignment) of the available COX3 X-ray crystal structure (*Rhodobacter sphaeroides* PDB: 1M56:C) with the corresponding COX3 3D structure of *Octopus vulgaris* (predicted in this study), using the PYMOL software version 1.5.0.4. Thus, we obtained a correspondence of the positively selected site numbering (assuming as reference the COX3 protein sequence of the *Octopus vulgaris*) to the COX3 sequence numbers of *Rhodobacter sphaeroides* species, which has described sites involved in proton coupling mechanisms (D-channel). (ii) We also performed a MUSCLE alignment (in the SEAVIEW software version 4.4.0) of the Cephalopoda COX3 dataset with its homolog from *Homo sapiens*. This approach allowed to establish a correspondence of described sites mutations related with diseases in humans, between *Homo sapiens* and cephalopods (*e*.*g*. *Octopus vulgaris*). Finally, we performed a profile alignment (using the GENEIOUS software version 5.6.7 *profile align* option) of the previous described alignments (i and ii), which allowed a correspondence of the sites among all the mentioned species. TREESAAP is mentioned when a site also presented amino acid properties positively selected (p-value < 0.001).
(DOCX)

S8 Table Likelihood ratio tests for PAML (CODEML) site models in 13 mitochondrial genes of the Cephalopoda dataset. (DOCX)

S9 Table Branch model test (CODEML) for the foreground cephalopod lineages. A to H indicate foreground-lineages selected according with the hypotheses displayed in Fig 3. Values are the ϖ for the foreground-lineages tested (A to H). The colours indicate the final hypothesis decision, according to the LRT between the null and the alternate model likelihoods, considering as significant a p-value < 0.05.
(DOCX)

S10 Table Branch-site model test (CODEML) for the foreground cephalopod lineages. A to H indicate foreground-lineages selected according with the hypotheses displayed in Fig 3. Values are the ω for the foreground-lineages tested (A to H). The colours indicate the final hypothesis decision, according to the LRT between the null and the alternate model likelihoods, considering as significant a p-value < 0.05.
(DOCX)
