# Supplementary material for: Adaptation of the Mitochondrial Genome in Cephalopods: Enhancing Proton Translocation Channels and the Subunit Interactions
Source: PLoS One. 2015 Aug 18;10(8):e0135405. doi: 10.1371/journal.pone.0135405 (PMC4540416; doi:10.1371/journal.pone.0135405)
Supplement: S1 Fig — At the top are the names of the mitochondrial genes. The arrow shapes indicate their orientation in the mitochondrial genomes. Each one of the duplicated genes included in our datasets are identified, based on their relative positions in the mitochondrial genomes, as left and right side duplicated genes, respectively. (DOCX) [file pone.0135405.s001.docx]

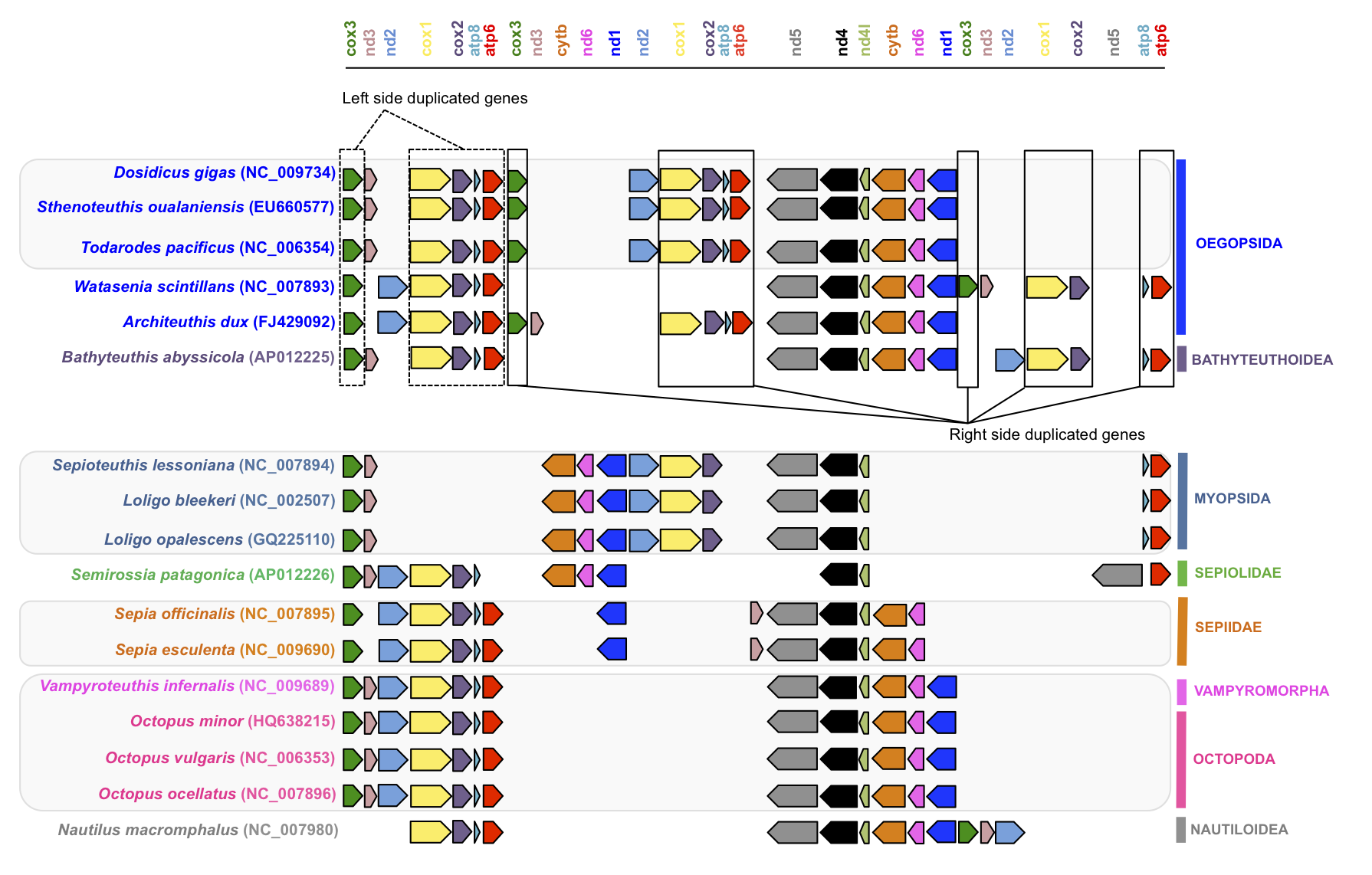


**S1 Fig. Mitochondrial genes included in Cephalopoda datasets.** At the top are the names of the mitochondrial genes. The arrow shapes indicate their orientation in the mitochondrial genomes. Each one of the duplicated genes included in our datasets are identified, based on their relative positions in the mitochondrial genomes, as left and right side duplicated genes, respectively.
