# Supplementary material for: Adaptation of the Mitochondrial Genome in Cephalopods: Enhancing Proton Translocation Channels and the Subunit Interactions
Source: PLoS One. 2015 Aug 18;10(8):e0135405. doi: 10.1371/journal.pone.0135405 (PMC4540416; doi:10.1371/journal.pone.0135405)

**S2 Fig. Architecture of the membrane domain of *Octopus vulgaris* Complex I.** At the bottom it is possible to observe a mitochondria scheme with the subunits composing the five mitochondrial OXPHOS complexes, named in roman numerals. The mitochondrial matrix is represented in light blue, the inner mitochondrial membrane in grey and the intermembrane space in light pink. At the top, a detailed view from the membrane plane of the Complex I. This was obtained by superimposition of the predicted 3D structures (I-TASSER) of the *Octopus vulgaris* (Common octopus) subunits with the entire Complex I from *Escherichia coli* (PDB code 3RKO). Subunits are colored as follows: ND5 - magenta, ND4 - navy blue, ND2 - yellow, ND6 - green, ND3 - light green, ND4L - purple and ND1 - beige. The remaining subunits are from the PDB code 3RKO. Helices that are probably involved in conformational changes are shown in red, orange and yellow. Helix HL is shown in hot pink (horizontal).


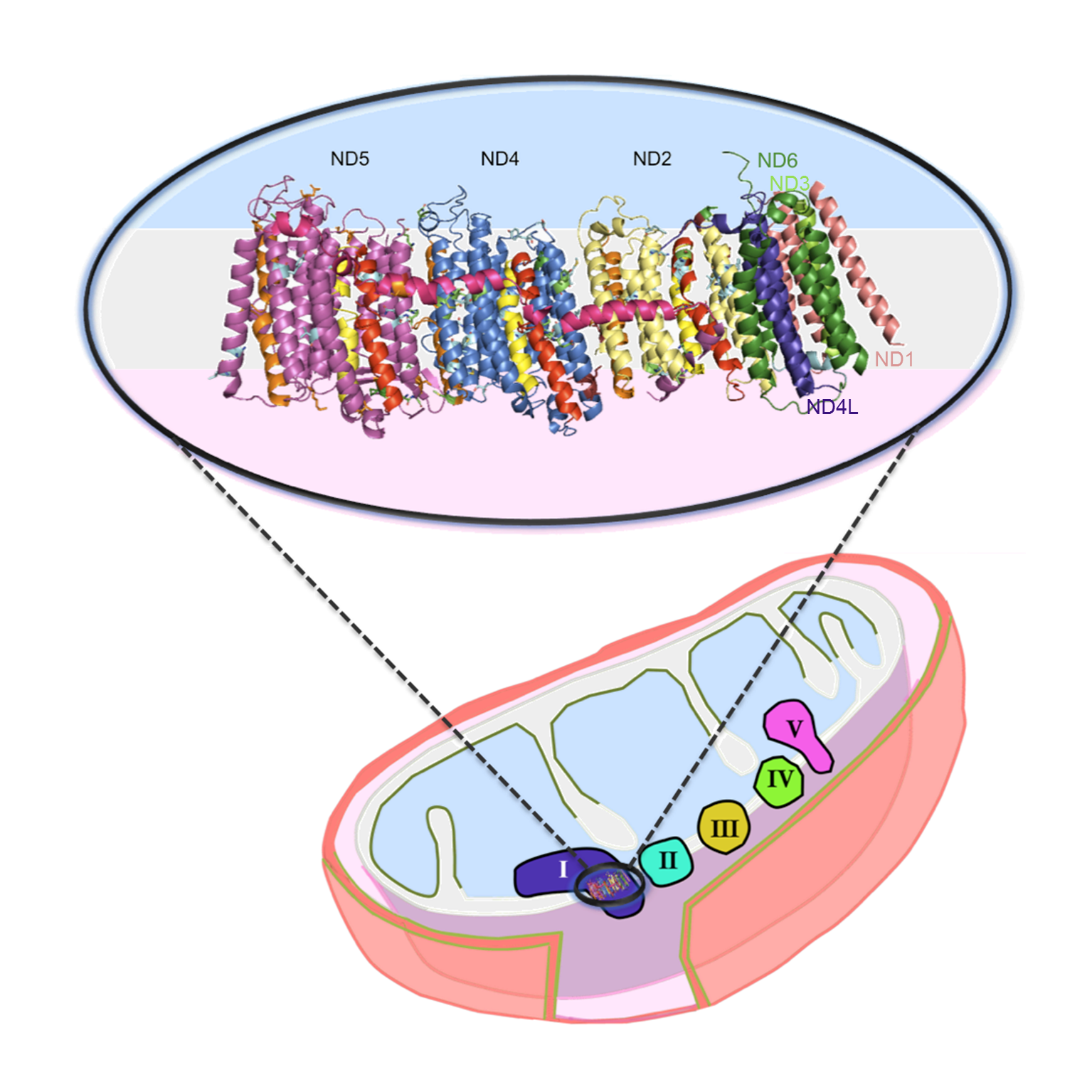

Supplement: S2 Fig — At the bottom it is possible to observe a mitochondria scheme with the subunits composing the five mitochondrial OXPHOS complexes, named in roman numerals. The mitochondrial matrix is represented in light blue, the inner mitochondrial membrane in grey and the intermembrane space in light pink. At the top, a detailed view from the membrane plane of the Complex I. This was obtained by superimposition of the predicted 3D structures (I-TASSER) of the Octopus vulgaris (Common octopus) subunits with the entire Complex I from Escherichia coli (PDB code 3RKO). Subunits are colored as follows: ND5—magenta, ND4—navy blue, ND2—yellow, ND6—green, ND3—light green, ND4L—purple and ND1—beige. The remaining subunits are from the PDB code 3RKO. Helices that are probably involved in conformational changes are shown in red, orange and yellow. Helix HL is shown in hot pink (horizontal). (DOCX) [file pone.0135405.s002.docx]
