# Supplementary material for: Adaptation of the Mitochondrial Genome in Cephalopods: Enhancing Proton Translocation Channels and the Subunit Interactions
Source: PLoS One. 2015 Aug 18;10(8):e0135405. doi: 10.1371/journal.pone.0135405 (PMC4540416; doi:10.1371/journal.pone.0135405)
Supplement: S3 Fig — Yellow circles show the sites that present high number of radically changing properties under positive destabilizing selection, according to TREESAAP. (DOCX) [file pone.0135405.s003.docx]

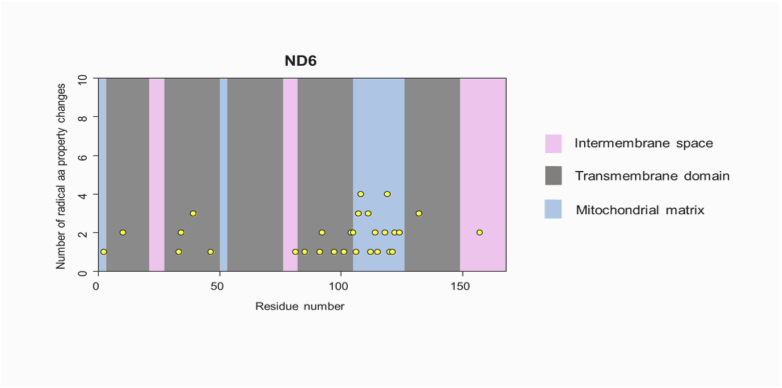


**S3 Fig. Topological assignment of ND6 subunit of Complex I.** Yellow circles show the sites that present high number of radically changing properties under positive destabilizing selection, according to TREESAAP.
