# Supplementary material for: Adaptation of the Mitochondrial Genome in Cephalopods: Enhancing Proton Translocation Channels and the Subunit Interactions
Source: PLoS One. 2015 Aug 18;10(8):e0135405. doi: 10.1371/journal.pone.0135405 (PMC4540416; doi:10.1371/journal.pone.0135405)
Supplement: S1 Table — In this table are provided the Accession numbers of all the Cephalopoda species contemplated in this study, as well as, more details about their: taxonomy (species scientific name, common name, Cephalopoda major taxa and family), habitat (ocean zones, temperatures and corresponding average depths) and metabolic rates. UNK—indicates missing data. (DOCX) [file pone.0135405.s005.docx]

**S1 Table. The Cephalopoda dataset in study.** In this table are provided the Accession numbers of all the Cephalopoda species contemplated in this study, as well as, more details about their: taxonomy (species scientific name, common name, Cephalopoda major taxa and family), habitat (ocean zones, temperatures and corresponding average depths) and metabolic rates. UNK - indicates missing data.

| **Accession number (Species)** | **Common name** | **Major taxa (Family)** | **Habitat** | **Average Ocean Depth (m)** | **Metabolic Rate** | **References** |
| --- | --- | --- | --- | --- | --- | --- |
|  |  |  |  |  |  |  |
| **NC_007980**  *Nautilus macromphalus* (Sowerby, 1849) | Bellybutton nautilus | **Nautiloidea** (Nautilidae) | Continental shelf and slope waters associated with coral reefs. Temperatures exceeding 25ºC may be lethal for these animals. | Deep (250) | **Low** | [1,2,3,4] |
| **NC_009689**   *Vampyroteuthis infernalis*  (Chun, 1903) | Vampire squid | **Vampyromorpha**  (Vampyroteuthidae) | Temperate and tropical oceans; low oxygen habitats - at oxygen saturations as low as 3%. | Deep (900) | **Very low** | [3,5,6,7,8,9] |
|  |  |  |  |  |  |  |
| **AP012226** *Semirossia patagonica* (Smith, 1881) | Patagonian bobtail squid | **Sepioidea**  (Sepiolidae) | Shallow coastal waters. | Intermediate (171) | **UNK** | [10] |
| **NC_009690**   *Sepia esculenta*  (Hoyle, 1885) | Golden cuttlefish | **Sepioidea** (Sepiidae) | Neritic species (mainly inner shelf), found on sand; sometimes burrowing into the  substrate. | Shallow (55) | **High** | [2,11] |
|  |  |  |  |  |  |  |
|  |  |  |  |  |  |  |
|  |  |  |  |  |  |  |
| **NC_007895**  *Sepia officinalis*  (Linnaeus, 1758) | Common cuttlefish |  | Continental shelf, predominantly on sandy or muddy substrates. Temperatures: 10ºC to 30ºC. | Shallow (100) |  | [2] |
|  |  |  |  |  |  |  |
|  |  |  |  |  |  |  |
|  |  |  |  |  |  |  |
| **HQ638215**  *Octopus minor (Sasaki, 1920)* | Korean common octopus | **Octopoda**  (Octopodidae) | Continental shelf. | Shallow (107) | **Intermediate** | [7,12] |
|  |  |  |  |  |  |  |
|  |  |  |  |  |  |  |
|  |  |  |  |  |  |  |
| **NC_007896** *Octopus ocellatus* (Gray, 1849) | Baby octopus |  | Bottom-living, shallow-water species. Optimal temperature range: 13ºC to 26ºC. | Shallow (32.5) |  |  |
| **NC_006353**  *Octopus vulgaris*  (Cuvier, 1797) | Common octopus |  | A benthic, neritic species occurring in a variety of habitats, such as rocks, and coral reefs. Tropical, subtropical and temperate waters. It is inactive in waters of 7°C and colder. | Shallow (51.5) |  |  |
| **NC_002507**  *Loligo bleekeri*  (Keferstein, 1866) | Spear squid | **Myopsida**  (Loliginidae) | Neritic species. Subtropical waters. Temperature: approximately 14ºC. | Shallow (75) | **Highest** | [7,12] |
|  |  |  |  |  |  |  |
| **GQ225110**   *Loligo opalescens* (Berry, 1911) | Opalescent inshore squid |  | Shallow coastal waters. Temperature: 10ºC to 16ºC. | Shallow (37.5) |  |  |
|  |  |  |  |  |  |  |
| **NC_007894**  *Sepioteuthis lessoniana*  (Lesson, 1830) | Bigfin reef squid |  | Neritic species. Temperature: 20ºC to 26ºC. | Shallow (50) |  |  |
|  |  |  |  |  |  |  |
| **AP012225**   *Bathyteuthis abyssicola*  (Hoyle, 1885) | Deepsea squid | **Bathyteuthoidea** (Bathyteuthidae) | Mesobathypelagic. Temperature: -0.039ºC to 20ºC. | Deep (1350) |  | [12] |
| **FJ429092**   *Architeuthis dux*  (Steenstrup, 1857) | Giant squid | **Oegopsida** (Architeuthidae) | Continental slopes. Temperature: 9ºC. | Deep (475) |  | [12,13] |
|  |  |  |  |  |  |  |
|  |  |  |  |  |  |  |
| **NC_009734**   *Dosidicus gigas* (D’Orbigny, 1835) | Jumbo flying squid | **Oegopsida**  (Ommastrephidae) | Temperature: 26 to 28ºC. | Deep (450) |  | [12] |
| **EU660577**  *Sthenoteuthis oualaniensis*  (Lesson, 1830) | Purpleback flying squid |  | Larvae are planktonic. Juveniles are often associated with the continental slope. Temperature: 7 to 28ºC. | Deep (500) |  | [4,12,14,15] |
| **NC_006354** *Todarodes pacificus*  (Steenstrup, 1880) | Japanese flying squid |  | Oceanic and neritic. Temperature: 5° to 27° C. | Deep (250) |  | [12] |
| **NC_007893**  *Watasenia scintillans*  (Berry, 1911) | Firefly squid | **Oegopsida**  (Enoploteuthidae) | Mesopelagic boundary species associated with shelf waters. Temperature: 3ºC to 15ºC. | Deep (850) |  | [12,16] |

**References:**

1. Hamada T, Deguchi Y, Nautilus JECoL, Obata I, Okutani T (1980) Nautilus Macromphalus in Captivity: Tokai University Press.
2. Jereb P, Roper CFE (2005) Cephalopods of the World: Chambered nautiluses and sepioids (Nautilidae, Sepiidae, Sepiolidae, Sepiadariidae, Idiosepiidae and Spirulidae): Food and Agriculture Organization of the United Nations.
3. Norman MD (2000) Cephalopods : a world guide : Pacific Ocean, Indian Ocean, Red Sea, Atlantic Ocean, Caribbean, Arctic, Antarctic / Mark Norman, Hackenheim, Germany: ConchBooks.
4. Norman MD, Reid AL (2000) A Guide to Squid, Cuttlefish and Octopuses of Australasia: Csiro Publishing.
5. Johnson B. (2000) Vampyroteuthis infernalis. Animal Diversity Web., Vol. 2012.
6. MarineBio. (2012) Vampire Squids, Vampyroteuthis infernalis ~ MarineBio.org., MarineBio Conservation Society. Web. , Vol. 2012.
7. Seibel BA (2007) On the depth and scale of metabolic rate variation: scaling of oxygen consumption rates and enzymatic activity in the Class Cephalopoda (Mollusca). J Exp Biol 210: 1-11
8. Seibel BA, Thuesen EV, Childress JJ (1998) Flight of the vampire: ontogenetic gait-transition in vampyroteuthis infernalis (Cephalopoda: vampyromorpha). J Exp Biol 201 2413-2424
9. Young RE. (2012) Vampyroteuthidae Thiele, in Chun, 1915. Vampyroteuthis infernalis Chun, 1903., The Tree of Life Web Project, Vol. 2012.
10. Barratt I, Allcock L. (2012a) Semirossia patagonica. IUCN 2012, IUCN Red List of Threatened Species, Vol. 2012.
11. Barratt I, Allcock L. (2012b) Sepia esculenta. IUCN, UCN Red List of Threatened Species, Vol. 2012.
12. Jereb P, Roper CFE (2010) Cephalopods of the world. An annotated and illustrated catalogue of cephalopod species known to date. Volume 2. Myopsid and Oegopsid Squids., Vol. 2, 4 edn. FAO Species Catalogue for Fishery Purposes.
13. Vaughan J. (2000) Architeuthis dux. Animal Diversity Web, Vol. 2012.
14. Roper CFE, Sweeney MJ, Nauen CE. (1984) Cephalopods of the world: an annotated and illustrated catalogue of species of interest to fisheries. United Nations Development Programme, FAO Fish Synopsis, Vol. 3, pp. 1-277.
15. Young RE, Vecchione M. (2012) Sthenoteuthis oualaniensis (Lesson, 1830). The Tree of Life Web Project, Vol. 2012.
16. Patel K, Pee D. (2011) Watasenia scintillans. Animal Diversity Web, Vol. 2012.
