# Supplementary material for: Adaptation of the Mitochondrial Genome in Cephalopods: Enhancing Proton Translocation Channels and the Subunit Interactions
Source: PLoS One. 2015 Aug 18;10(8):e0135405. doi: 10.1371/journal.pone.0135405 (PMC4540416; doi:10.1371/journal.pone.0135405)
Supplement: S2 Table — Green—highlights synonymous substitutions. Red—highlights non-synonymous substitutions. (=) means that both the genes are equal copies, without any nucleotide change. Nucleotides are abbreviated with the one-letter codes, as well as the amino acids. Example: Dosidicus gigas—considering the cox3 gene, A568G means that in the nucleotide number 568 an adenine (A) was changed by a guanine (G) nucleotide, resulting in a non-synonymous mutation in the codon number 190. At the amino acid level it is reflected in the change of an isoleucine (I) by a valine (V) amino acid. The substitution number position has as reference the gene that first appears in the mt genome (left side), comparing it with the second copy positioned after (right side duplicated gene). Oegopsida—without cornea. Myopsida—with cornea. (DOCX) [file pone.0135405.s006.docx]

**S2 Table. Mutations in the duplicated mitochondrial genes detected in our Cephalopoda dataset.** Green - highlights synonymous substitutions. Red - highlights non-synonymous substitutions. (=) means that both the genes are equal copies, without any nucleotide change. Nucleotides are abbreviated with the one-letter codes, as well as the amino acids. Example: *Dosidicus gigas* - considering the cox3 gene, A568G means that in the nucleotide number 568 an adenine (A) was changed by a guanine (G) nucleotide, resulting in a non-synonymous mutation in the codon number 190. At the amino acid level it is reflected in the change of an isoleucine (I) by a valine (V) amino acid. The substitution number position has as reference the gene that first appears in the mt genome (left side), comparing it with the second copy positioned after (right side duplicated gene). Oegopsida - without cornea. Myopsida - with cornea.

|  |  |  |  | **COX1** | | |  | **COX2** | | |  | **COX3** | | |  | **ATP6** | | |  | **ATP8** | | |
| --- | --- | --- | --- | --- | --- | --- | --- | --- | --- | --- | --- | --- | --- | --- | --- | --- | --- | --- | --- | --- | --- | --- |
| **Taxonomic groups** |  | **Species** |  | **Codon** | **Nucleotide substitution** | **Amino acid** |  | **Codon** | **Nucleotide substitution** | **Amino acid** |  | **Codon** | **Nucleotide substitution** | **Amino acid** |  | **Codon** | **Nucleotide substitution** | **Amino acid** |  | **Codon** | **Nucleotide substitution** | **Amino acid** |
| Oegopsida |  | *Dosidicus gigas* |  | = | | |  | = | | |  | 190 | A568G | I🡺V |  | = | | |  | = | | |
|  |  |  |  |  |  |  |  |  |  |  |  | 219 | A657G | R |  |  |  |  |  |  |  |  |
|  |  |  |  |  |  |  |  |  |  |  |  | 248 | T742C | L |  |  |  |  |  |  |  |  |
|  |  | *Sthenoteuthis oualaniensis* |  | 30 | A90G | L |  | 40 | C120T | Y |  | = | | |  | 111 | G333A | L |  | = | | |
|  |  |  |  | 124 | A372G | T |  |  |  |  |  |  |  |  |  |  |  |  |  |  |  |  |
|  |  |  |  | 150 | C450T | A |  |  |  |  |  |  |  |  |  |  |  |  |  |  |  |  |
|  |  |  |  | 183 | A549G | W |  |  |  |  |  |  |  |  |  |  |  |  |  |  |  |  |
|  |  |  |  | 349 | G1047A | G |  |  |  |  |  |  |  |  |  |  |  |  |  |  |  |  |
|  |  |  |  | 490 | G1470A | E |  |  |  |  |  |  |  |  |  |  |  |  |  |  |  |  |
|  |  | *Todarodes pacificus* |  | = | | |  | = | | |  | 204 | T612C | L |  | 109 | G325C | V🡺L |  | = | | |
|  |  | *Watasenia scintillans* |  | 49 | G147A | Q |  | 215 | C645T | N |  | 38 | T114A | I🡺M |  | 70 | C210T | G |  | = | | |
|  |  |  |  |  |  |  |  |  |  |  |  | 161 | T482G | M🡺S |  | 197 | A589G | M🡺V |  |  |  |  |
|  |  |  |  |  |  |  |  |  |  |  |  | 258 | G774A | G |  |  |  |  |  |  |  |  |
|  |  | *Architeuthis dux* |  | 224 | C672T | D |  | = | | |  | 76 | T228C | G |  | = | | |  | = | | |
| Bathyteuthoidea |  | *Bathyteuthis abyssicola* |  | 267 | C800T | A🡺V |  | 52 | G155A A156C | W🡺Y |  | Without duplicated genes | | |  | = | | |  | = | | |
|  |  |  |  |  |  |  |  | 112 | A334G | N🡺D |  |  |  |  |  |  |  |  |  |  |  |  |
| Myopsida |  | *Sepioteuthis lessoniana* |  | Without duplicated genes | | | | | | | | | | | | | | | | | | |
|  |  | *Loligo bleekeri* |  |  |  |  |  |  |  |  |  |  |  |  |  |  |  |  |  |  |  |  |
|  |  | *Loligo opalescens* |  |  |  |  |  |  |  |  |  |  |  |  |  |  |  |  |  |  |  |  |
