# Supplementary material for: Adaptation of the Mitochondrial Genome in Cephalopods: Enhancing Proton Translocation Channels and the Subunit Interactions
Source: PLoS One. 2015 Aug 18;10(8):e0135405. doi: 10.1371/journal.pone.0135405 (PMC4540416; doi:10.1371/journal.pone.0135405)
Supplement: S3 Table — The sites identified as positively selected by branch-site analyses (CODEML and MEME: p-value < 0.05) were mapped in the Cephalopoda ND5 protein sequence alignment (Cephalopoda ND5 dataset: obtained through the translation of the respective MUSCLE codon based CDS alignment, performed in SEAVIEW software version 4.4.0). Then, we performed a profile alignment (using the GENEIOUS software version 5.6.7 profile align option) of the (i) Cephalopoda ND5 dataset (17 species) with the (ii) structure-based alignment (containing 30 representative species from all kingdoms of life) of the ND5 subunit from the study of Efremov & Sazanov (2011) [1]. Thus, we obtained a correspondence of the positively selected sites numbering (assuming as reference the ND5 protein sequence of the Octopus vulgaris) to the sequence numbers of species (Escherichia coli and Homo sapiens) with described residues involved in interactions between subunits, forming proton translocation channels and with associated mutations. TREESAAP is mentioned when a site also presented amino acid properties positively selected (p-value < 0.001). (DOCX) [file pone.0135405.s007.docx]

**S3 Table. Homology analyses of the ND5 subunit.** The sites identified as positively selected by branch-site analyses (CODEML and MEME: p-value < 0.05) were mapped in the Cephalopoda ND5 protein sequence alignment (Cephalopoda ND5 dataset: obtained through the translation of the respective MUSCLE codon based CDS alignment, performed in SEAVIEW software version 4.4.0). Then, we performed a profile alignment (using the GENEIOUS software version 5.6.7 profile align option) of the (i) Cephalopoda ND5 dataset (17 species) with the (ii) structure-based alignment (containing 30 representative species from all kingdoms of life) of the ND5 subunit from the study of Efremov & Sazanov (2011) [1]. Thus, we obtained a correspondence of the positively selected sites numbering (assuming as reference the ND5 protein sequence of the *Octopus vulgaris*) to the sequence numbers of species (*Escherichia coli* and *Homo sapiens*) with described residues involved in interactions between subunits, forming proton translocation channels and with associated mutations. TREESAAP is mentioned when a site also presented amino acid properties positively selected (p-value < 0.001).

| ***Octopus vulgaris* (Common octopus - NC_006353)** | ***Escherichia coli* (Bacterium - NuoL: 3RKO:B)** | ***Homo sapiens* (Human - ADB78261.1)** | **Features** | **References** |
| --- | --- | --- | --- | --- |
| K3 | E27 | _ | CODEML and TREESAAP | This study |
| V17 | A41 | T43 | CODEML and TREESAAP | This study |
| S38 | L64 | N65 | CODEML | This study |
| I41 | W67 | W68 | Interactions between NuoM (ND4) and NuoL (ND5) subunits | [1] |
| C74 | H100 | M101 | Proton channel 1: main link to cytoplasm (mitochondrial matrix); CODEML | [1] and This study |
| V93 | Y119 | Y120 | Proton channel 1: Possible link at the interface of the NuoL (ND5) and NuoM (ND4) subunits; TREESAAP | [1] and This study |
| L94 | T120 | L121 | Proton channel 1: main link to cytoplasm (mitochondrial matrix) | [1] |
| P108 | D134N | N135 | Mutation: 70% proton pumping | [1,2] |
| D118 | E144 | E145 | Proton channel 1: amino acid lining the main cavity | [1] |
| F125 | Y151 | F152 | Proton channel 1: Possible link at the interface of the NuoL (ND5) and NuoM (ND4) subunits | [1] |
| Q132 | Y158 | Y159 | Interactions between NuoM (ND4) and NuoL (ND5) subunits | [1] |
| L142 | K169C/E/R | Q170 | Mutation: proton pumping decreased | [1,3] |
| T143 | A170 | A171V | LHON | [1,4] |
| N147 | T174 | N175 | Proton channel 1: amino acid lining the main cavity | [1] |
| S159 | I187 | W188 | CODEML | This study |
| M161 | Y189 | I190 | Interactions between NuoM (ND4) and NuoL (ND5) subunits | [1] |
| S162 | N190 | L191 | Interactions between NuoM (ND4) and NuoL (ND5) subunits | [1] |
| S199 | Q236K | H230 | Mutation: proton pumping decreased | [1,3] |
| S218 | A255 | S249 | Connection of proton channel 1 with proton channel 2 | [1] |
| V239 | T276 | S270 | CODEML | This study |
| V281 | Y318 | L312 | Proton channel 2: main cavity | [1] |
| K289 | Q326 | N320 | CODEML | This study |
| D327 | K364 | K358 | CODEML and TREESAAP | This study |
| S392 | T429 | S423 | Proton channel 2: main cavity | [1] |
| D415 | G450 | N446 | MEME | This study |
| C427 | I462 | A458T | LHON | [1,5] |
| K455 | E494 | K487 | Proton channel 2: link to periplasm (intermembrane space) | [1] |
| G475 | S521 | N509 | CODEML and TREESAAP | This study |
| S497 | W543 | S531 | CODEML | This study |
| M507 | F553 | G541 | Interactions between NuoM (ND4) and NuoL (ND5) subunits; TREESAAP | [1] and This study |
| K516 | D563 | L551 | Interactions between NuoM (ND4) and NuoL (ND5) subunits | [1] |
| E524 | L565 | E559 | Interactions between NuoM (ND4) and NuoL (ND5) subunits | [1] |
| M525 | N566 | K560 | Interactions between NuoM (ND4) and NuoL (ND5) subunits | [1] |
| F550 | Y594 | Y587 | Interactions between NuoN (ND2) and NuoL (ND5) subunits | [1] |
| N553 | S597 | S590 | Interactions between NuoN (ND2) and NuoL (ND5) subunits | [1] |
| **(i) Cephalopoda ND5 dataset [from this study]** | **(ii) Structure-based alignment from [1]** | |  |  |
| **Profile alignment** | | |  |  |

**References:**

1. Efremov RG, Sazanov LA (2011) Structure of the membrane domain of respiratory complex I. Nature 476: 414-420.

2. Nakamaru-Ogiso E, Kao MC, Chen H, Sinha SC, Yagi T, et al. (2010) The membrane subunit NuoL(ND5) is involved in the indirect proton pumping mechanism of Escherichia coli complex I. J Biol Chem 285: 39070-39078.

3. Michel J, DeLeon-Rangel J, Zhu S, Van Ree K, Vik SB (2011) Mutagenesis of the L, M, and N subunits of Complex I from Escherichia coli indicates a common role in function. PLoS One 6: e17420.

4. Mayorov V, Biousse V, Newman NJ, Brown MD (2005) The role of the ND5 gene in LHON: characterization of a new, heteroplasmic LHON mutation. Ann Neurol 58: 807-811.

5. Brown MD, Voljavec AS, Lott MT, Torroni A, Yang CC, et al. (1992) Mitochondrial DNA complex I and III mutations associated with Leber's hereditary optic neuropathy. Genetics 130: 163-173.
