# Supplementary material for: Adaptation of the Mitochondrial Genome in Cephalopods: Enhancing Proton Translocation Channels and the Subunit Interactions
Source: PLoS One. 2015 Aug 18;10(8):e0135405. doi: 10.1371/journal.pone.0135405 (PMC4540416; doi:10.1371/journal.pone.0135405)
Supplement: S4 Table — The sites identified as positively selected by branch-site analyses (MEME: p-value < 0.05) were mapped in the Cephalopoda ND6 protein sequence alignment (Cephalopoda ND6 dataset: obtained through the translation of the respective MUSCLE codon based CDS alignment, performed in SEAVIEW software version 4.4.0). Then, we performed a profile alignment (using the GENEIOUS software version 5.6.7 profile align option) of the (i) Cephalopoda ND6 dataset (17 species) with the (ii) structure-based alignment (containing 30 representative species from all kingdoms of life) of the ND6 subunit from the study of Efremov & Sazanov (2011) [1]. Thus, we obtained a correspondence of the positively selected sites numbering (assuming as reference the ND6 protein sequence of the Octopus vulgaris) to the sequence numbers of species (Escherichia coli and Homo sapiens) with described residues involved in interactions between subunits, forming proton translocation channels and with associated mutations. TREESAAP is mentioned when a site also presented amino acid properties positively selected (p-value < 0.001). (DOCX) [file pone.0135405.s008.docx]

**S4 Table. Homology analyses of the ND6 subunit.** The sites identified as positively selected by branch-site analyses (MEME: p-value < 0.05) were mapped in the Cephalopoda ND6 protein sequence alignment (Cephalopoda ND6 dataset: obtained through the translation of the respective MUSCLE codon based CDS alignment, performed in SEAVIEW software version 4.4.0). Then, we performed a profile alignment (using the GENEIOUS software version 5.6.7 profile align option) of the (i) Cephalopoda ND6 dataset (17 species) with the (ii) structure-based alignment (containing 30 representative species from all kingdoms of life) of the ND6 subunit from the study of Efremov & Sazanov (2011) [1]. Thus, we obtained a correspondence of the positively selected sites numbering (assuming as reference the ND6 protein sequence of the *Octopus vulgaris*) to the sequence numbers of species (*Escherichia coli* and *Homo sapiens*) with described residues involved in interactions between subunits, forming proton translocation channels and with associated mutations. TREESAAP is mentioned when a site also presented amino acid properties positively selected (p-value < 0.001).

| ***Octopus vulgaris* (Common octopus - NC_006353)** | ***Escherichia coli* (Bacterium - NuoJ: P0AFE0)** | ***Homo sapiens* (Human - P03923)** | **Features** | **References** |
| --- | --- | --- | --- | --- |
| Y6 | I7 | L7 | MEME | This study |
| L24 | V26 | I26M | Surface, possibly near Q-site; LHON and severe complex I deficiency | [1,2] |
| L32 | I34 | V34 | MEME | This study |
| V33 | S35 | S35 | Main channel 4; TREESAAP | [1] and This study |
| S34 | L36 | G36S | Near channel 4; LHON; TREESAAP | [1,3] and This study |
| L53 | E55 | V55 | Main channel 4 | [1] |
| V58 | A60 | L60S | TM3, near channel 4; LHON | [1,4] |
| L62 | M64V | M64V | TM3, near channel 4; LHON | [1,5,6] |
|  | M64C | M64C | TM3, near channel 5 | [1,7] |
|  | M64I | M64I | TM3, near channel 6; LHON | [1,5,8] |
| S70 | M72V | A72V | TM3, interface with NuoA (ND3)/ NuoH (ND1)  LHON/ Leigh disease/ dystonia and Complex I deficiency | [1,7,9] |
|  | M72A | A72 | TM3, interface with NuoA (ND3)/ NuoH (ND1) | [1,7] |
|  | M72C |  |  |  |
| I72 | L74 | A74V | TM3, near surface | [1] |
| K113 | Q117 | E128 | MEME | This study |
| F119 | P123 | L134 | MEME and TREESAAP | This study |
| F139 | E142 | V154 | Alternative link to periplasm (intermembrane space) of channel 4 | [1] |
| V142 | S145 | G157 | Alternative link to periplasm (intermembrane space) of channel 4 | [1] |
| **(i) Cephalopoda ND6 dataset [from this study]** | **(ii) Structure-based alignment from [1]** | |  |  |
| **Profile alignment** | | |  |  |

**References:**

1. Efremov RG, Sazanov LA (2011) Structure of the membrane domain of respiratory complex I. Nature 476: 414-420.

2. De Vries DD, Went LN, Bruyn GW, Scholte HR, Hofstra RM, et al. (1996) Genetic and biochemical impairment of mitochondrial complex I activity in a family with Leber hereditary optic neuropathy and hereditary spastic dystonia. Am J Hum Genet 58: 703-711.

3. Wissinger B, Besch D, Baumann B, Fauser S, Christ-Adler M, et al. (1997) Mutation analysis of the ND6 gene in patients with Lebers hereditary optic neuropathy. Biochem Biophys Res Commun 234: 511-515.

4. Chinnery PF, Brown DT, Andrews RM, Singh-Kler R, Riordan-Eva P, et al. (2001) The mitochondrial ND6 gene is a hot spot for mutations that cause Leber's hereditary optic neuropathy. 209-218 p.

5. Kao MC, Di Bernardo S, Nakamaru-Ogiso E, Miyoshi H, Matsuno-Yagi A, et al. (2005) Characterization of the membrane domain subunit NuoJ (ND6) of the NADH-quinone oxidoreductase from Escherichia coli by chromosomal DNA manipulation. Biochemistry 44: 3562-3571.

6. Carelli V, Ghelli A, Bucchi L, Montagna P, De Negri A, et al. (1999) Biochemical features of mtDNA 14484 (ND6/M64V) point mutation associated with Leber's hereditary optic neuropathy. Ann Neurol 45: 320-328.

7. Patsi J, Kervinen M, Finel M, Hassinen IE (2008) Leber hereditary optic neuropathy mutations in the ND6 subunit of mitochondrial complex I affect ubiquinone reduction kinetics in a bacterial model of the enzyme. Biochem J 409: 129-137.

8. Valentino ML, Avoni P, Barboni P, Pallotti F, Rengo C, et al. (2002) Mitochondrial DNA nucleotide changes C14482G and C14482A in the ND6 gene are pathogenic for Leber's hereditary optic neuropathy. Ann Neurol 51: 774-778.

9. Kirby DM, Kahler SG, Freckmann ML, Reddihough D, Thorburn DR (2000) Leigh disease caused by the mitochondrial DNA G14459A mutation in unrelated families. Ann Neurol 48: 102-104.
