# Supplementary material for: Adaptation of the Mitochondrial Genome in Cephalopods: Enhancing Proton Translocation Channels and the Subunit Interactions
Source: PLoS One. 2015 Aug 18;10(8):e0135405. doi: 10.1371/journal.pone.0135405 (PMC4540416; doi:10.1371/journal.pone.0135405)
Supplement: S5 Table — The sites identified as positively selected by branch-site analyses (CODEML and MEME: p-value < 0.05) were mapped in the Cephalopoda CYTB protein sequence alignment (Cephalopoda CYTB dataset: obtained through the translation of the respective MUSCLE codon based CDS alignment, performed in SEAVIEW software version 4.4.0). (i) Then, we performed the superimposition (structure-based alignment) of the available CYTB X-ray crystal structures (Bos taurus PDB 1PPJ:C and Saccharomyces cerevisiae PDB 1P84:C) with the corresponding CYTB 3D structure of Octopus vulgaris (predicted in this study), using the PYMOL software version 1.5.0.4. Thus, we obtained a correspondence of the positively selected sites numbering (assuming as reference the CYTB protein sequence of the Octopus vulgaris) to the sequence numbers of species (Bos taurus and Saccharomyces cerevisiae), with relevant functional binding sites described. (ii) We also performed a MUSCLE alignment (in the SEAVIEW software version 4.4.0) of the Cephalopoda CYTB dataset with its homolog from Homo sapiens. This approach allowed to establish a correspondence of sites with described mutations causing diseases in humans, between Homo sapiens and cephalopods (e.g. Octopus vulgaris). Finally, we performed a profile alignment (using the GENEIOUS software version 5.6.7 profile align option) of the previous described alignments (i and ii), which allowed a correspondence of the sites among all the mentioned species. (DOCX) [file pone.0135405.s009.docx]

**S5 Table. Homology analyses of the CYTB subunit.** The sites identified as positively selected by branch-site analyses (CODEML and MEME: p-value < 0.05) were mapped in the Cephalopoda CYTB protein sequence alignment (Cephalopoda CYTB dataset: obtained through the translation of the respective MUSCLE codon based CDS alignment, performed in SEAVIEW software version 4.4.0). (i) Then, we performed the superimposition (structure-based alignment) of the available CYTB X-ray crystal structures (*Bos taurus* PDB 1PPJ:C and *Saccharomyces cerevisiae* PDB 1P84:C) with the corresponding CYTB 3D structure of *Octopus vulgaris* (predicted in this study), using the PYMOL software version 1.5.0.4. Thus, we obtained a correspondence of the positively selected sites numbering (assuming as reference the CYTB protein sequence of the *Octopus vulgaris*) to the sequence numbers of species (*Bos taurus* and *Saccharomyces cerevisiae*), with relevant functional binding sites described. (ii) We also performed a MUSCLE alignment (in the SEAVIEW software version 4.4.0) of the Cephalopoda CYTB dataset with its homolog from *Homo sapiens*. This approach allowed to establish a correspondence of sites with described mutations causing diseases in humans, between *Homo sapiens* and cephalopods (e.g. *Octopus vulgaris*). Finally, we performed a profile alignment (using the GENEIOUS software version 5.6.7 *profile align* option) of the previous described alignments (i and ii), which allowed a correspondence of the sites among all the mentioned species.

| ***Bos taurus* (Bovine PDB: 1PPJ:C)** | ***Saccharomyces cerevisiae* (Yeast PDB: 1P84:C)** | **Cephalopoda CYTB dataset** | ***Homo sapiens* (Human - P00156)** | **Features** | **References** |
| --- | --- | --- | --- | --- | --- |
|  |  | ***Octopus vulgaris* (Common octopus - NC_006353)** |  |  |  |
| A17 | Y16 | A18 | S17 | Qi site | [1,2], PDB: 1PPJ chain C |
| I27 | I26 | L28 | I27 | Qi site | [1,2], PDB: 1PPJ chain C |
| W31 | W30 | W32 | W31 | b_H_ binding site | [1,2], PDB: 1PPJ chain C |
| G34 | G33 | G35 | G34 | b_H_ binding site | [1,2], PDB: 1PPJ chain C |
|  |  |  | G34S | Exercise intolerance in humans | [3] |
| S35 | S34 | S36 | S35 | Qi site | [1,2], PDB: 1PPJ chain C |
| Q44 | Q43 | Q45 | Q44 | b_L_ binding site | [1,2], PDB: 1PPJ chain C |
| G48 | G47 | G49 | G48 | b_L_ binding site | [1,2], PDB: 1PPJ chain C |
| L49 | I48 | I50 | L49 | b_L_ binding site | [1,2], PDB: 1PPJ chain C |
| L51 | M50 | L52 | L51 | b_L_ binding site | [1,2], PDB: 1PPJ chain C |
| T61 | L60 | Y62 | T61 | CODEML | This study |
| H83 | H82 | H84 | H83 | b_L_ binding site | [1,2], PDB: 1PPJ chain C |
| F90 | F89 | F91 | F90 | b_L_ binding site | [1,2], PDB: 1PPJ chain C |
| H97 | H96 | H98 | H97 | b_H_ binding site | [1,2], PDB: 1PPJ chain C |
| V98 | M97 | I99 | I98 | b_H_ binding site | [1,2], PDB: 1PPJ chain C |
| R100 | K99 | R101 | R100 | b_H_ binding site | [1,2], PDB: 1PPJ chain C |
| S106 | S105 | S107 | S106 | b_H_ binding site | [1,2], PDB: 1PPJ chain C |
| W113 | W114 | W114 | W113 | b_H_ binding site | [1,2], PDB: 1PPJ chain C |
|  |  |  | W113STOP | Exercise intolerance in humans | [3] |
| G116 | G117 | G117 | G116 | b_H_ binding site | [1,2], PDB: 1PPJ chain C |
| V117 | V118 | V118 | I117 | b_H_ binding site | [1,2], PDB: 1PPJ chain C |
| F128 | F129 | F129 | F128 | Involved in docking ubiquinol into the Qo pocket | [4] |
| M129 | L130 | V130 | M129 | Qo site | [1,2], PDB: 1PPJ chain C |
| Y131 | Y132 | Y132 | Y131 | Involved in proton conduction linked to ubiquinol oxidation | [4] |
| W135 | Y136 | W136 | W135STOP | Exercise intolerance in humans | [3] |
| M138 | M139 | M139 | M138 | Qo site | [1,2], PDB: 1PPJ chain C |
| W141 | W142 | W142 | W141STOP | Exercise intolerance in humans | [3] |
| G142 | G143 | G143 | G142STOP | Exercise intolerance in humans | [3] |
|  |  |  | G142 | Qo site | [1,2], PDB: 1PPJ chain C |
| V145 | V146 | V146 | V145 | Qo site | [1,2], PDB: 1PPJ chain C |
| I146 | I147 | I147 | I146 | Qo site | [1,2], PDB: 1PPJ chain C |
| S151 | S152 | S152 | S151P | Exercise intolerance in humans | [3] |
| L160 | I161 | L161 | L160 | Qo site | [1,2], PDB: 1PPJ chain C |
| V161 | V162 | V162 | V161 | Qo site | [1,2], PDB: 1PPJ chain C |
| H182 | H183 | H183 | H182 | b_L_ binding site | [1,2], PDB: 1PPJ chain C |
| F183 | Y184 | F184 | F183 | b_L_ binding site | [1,2], PDB: 1PPJ chain C |
| P186 | P187 | P187 | P186 | b_L_ binding site | [1,2], PDB: 1PPJ chain C |
| M194 | I195 | I195 | T194 | Qi site | [1,2], PDB: 1PPJ chain C |
| H196 | H197 | H197 | H196 | b_H_ binding site | [1,2], PDB: 1PPJ chain C |
| L197 | L198 | F198 | L197 | b_H_ binding site | [1,2], PDB: 1PPJ chain C |
| L200 | L201 | L201 | L200 | b_H_ binding site | [1,2], PDB: 1PPJ chain C |
| S205 | S206 | S206 | S205 | b_H_ binding site | [1,2], PDB: 1PPJ chain C |
| N206 | S207 | N207 | N206 | b_H_ binding site | [1,2], PDB: 1PPJ chain C |
| I211 | I212 | L212 | I211 | MEME | This study |
| D228 | D229 | D229 | D228 | Qi site | [1,2], PDB: 1PPJ chain C |
| P270 | P271 | P271 | P279 | Qo site | [1,2], PDB: 1PPJ chain C |
| E271 | E272 | E272 | E271 | Involved in proton conduction linked to ubiquinol oxidation | [4] |
|  |  |  |  | Qo site | [1,2], PDB: 1PPJ chain C |
| Y278 | Y279 | Y279 | Y278 | Involved in docking ubiquinol into the Qo pocket | [4] |
|  |  |  |  | Qo site | [1,2], PDB: 1PPJ chain C |
| G290 | G291 | G291 | G290D | Exercise intolerance in humans | [3] |
| R318 | K319 | N319 | R318P | Exercise intolerance in humans | [3] |
| W326 | F327 | W327 | W326STOP | Exercise intolerance in humans | [3] |
| V329 | V330 | I330 | A329 | MEME | This study |
| G339 | G340 | G340 | G339STOP | Exercise intolerance in humans | [3] |
|  |  | **(ii) MUSCLE alignment** | |  |  |
| **(i) Superimposition (Structure-based alignment)** | | |  |  |  |
| **Profile alignment** | | | |  |  |

**References:**

1. Huang LS, Cobessi D, Tung EY, Berry EA (2005) Binding of the respiratory chain inhibitor antimycin to the mitochondrial bc1 complex: a new crystal structure reveals an altered intramolecular hydrogen-bonding pattern. J Mol Biol 351: 573-597.

2. Xia D, Yu CA, Kim H, Xia JZ, Kachurin AM, et al. (1997) Crystal structure of the cytochrome bc1 complex from bovine heart mitochondria. Science 277: 60-66.

3. Meunier B, Fisher N, Ransac S, Mazat JP, Brasseur G (2013) Respiratory complex III dysfunction in humans and the use of yeast as a model organism to study mitochondrial myopathy and associated diseases. Biochim Biophys Acta 1827: 1346-1361.

4. Wenz T, Covian R, Hellwig P, Macmillan F, Meunier B, et al. (2007) Mutational analysis of cytochrome b at the ubiquinol oxidation site of yeast complex III. J Biol Chem 282: 3977-3988.
