# Supplementary material for: Adaptation of the Mitochondrial Genome in Cephalopods: Enhancing Proton Translocation Channels and the Subunit Interactions
Source: PLoS One. 2015 Aug 18;10(8):e0135405. doi: 10.1371/journal.pone.0135405 (PMC4540416; doi:10.1371/journal.pone.0135405)
Supplement: S6 Table — The site identified as positively selected by branch-site analyses (MEME: p-value < 0.05) was mapped in the Cephalopoda COX2 protein sequence alignment (Cephalopoda COX2 dataset: obtained through the translation of the respective MUSCLE codon based CDS alignment; performed in SEAVIEW software version 4.4.0). (i) Then, we performed the superimposition (structure-based alignment) of the available COX2 X-ray crystal structure (Bos taurus PDB 1V54:B) with the corresponding COX2 3D structure of Octopus vulgaris (predicted in this study), using the PYMOL software version 1.5.0.4. Thus, we obtained a correspondence of the positively selected site numbering (assuming as reference the COX2 protein sequence of the Octopus vulgaris) to the COX2 sequence numbers of Bos taurus species, which has described sites involved in proton coupling mechanisms (K-channel) and functional binding sites. (ii) We also performed a MUSCLE alignment (in the SEAVIEW software version 4.4.0) of the Cephalopoda COX2 dataset with its homolog from Homo sapiens. This approach allowed to establish a correspondence of described sites mutations related with exercise intolerance in humans, between Homo sapiens and cephalopods (e.g. Octopus vulgaris). Finally, we performed a profile alignment (using the GENEIOUS software version 5.6.7 profile align option) of the previous described alignments (i and ii), which allowed a correspondence of the sites among all the mentioned species. TREESAAP is mentioned when a site also presented amino acid properties positively selected (p-value < 0.001). (DOCX) [file pone.0135405.s010.docx]

**S6 Table. Homology analyses of the COX2 subunit.** The site identified as positively selected by branch-site analyses (MEME: p-value < 0.05) was mapped in the Cephalopoda COX2 protein sequence alignment (Cephalopoda COX2 dataset: obtained through the translation of the respective MUSCLE codon based CDS alignment; performed in SEAVIEW software version 4.4.0). (i) Then, we performed the superimposition (structure-based alignment) of the available COX2 X-ray crystal structure (*Bos taurus* PDB 1V54:B) with the corresponding COX2 3D structure of *Octopus vulgaris* (predicted in this study), using the PYMOL software version 1.5.0.4. Thus, we obtained a correspondence of the positively selected site numbering (assuming as reference the COX2 protein sequence of the *Octopus vulgaris*) to the COX2 sequence numbers of *Bos taurus* species, which has described sites involved in proton coupling mechanisms (K-channel) and functional binding sites. (ii) We also performed a MUSCLE alignment (in the SEAVIEW software version 4.4.0) of the Cephalopoda COX2 dataset with its homolog from *Homo sapiens*. This approach allowed to establish a correspondence of described sites mutations related with exercise intolerance in humans, between *Homo sapiens* and cephalopods (e.g. *Octopus vulgaris*). Finally, we performed a profile alignment (using the GENEIOUS software version 5.6.7 *profile align* option) of the previous described alignments (i and ii), which allowed a correspondence of the sites among all the mentioned species. TREESAAP is mentioned when a site also presented amino acid properties positively selected (p-value < 0.001).

| ***Bos taurus*  (Bovine - PDB: 1V54:B)** | **Cephalopoda COX2 dataset** | ***Homo sapiens* (Human - P00403)** | **Features** | **References** |
| --- | --- | --- | --- | --- |
|  | ***Octopus vulgaris* (Common octopus - NC_006353)** |  |  |  |
| T13 | N13 | T13 | MEME and TREESAAP | This study |
| M29 | F29 | M29K | Mutation that causes exercise intolerance in humans | [2] |
| E62 | E62 | E62 | K-channel | [1] |
| W65 | W65 | W65 | K-channel | [1] |
| T66 | T66 | T66 | K-channel | [1] |
| H161 | H161 | H161 | Cu_A_ metal binding site | UniProt (http://www.uniprot.org/uniprot/P00403) |
| C196 | C196 | C196 | Cu_A_ metal binding site | UniProt (http://www.uniprot.org/uniprot/P00403) |
| E198 | E198 | E198 | Mg^2+^ metal binding site | [1] |
| C200 | C200 | C200 | Cu_A_ metal binding site | UniProt (http://www.uniprot.org/uniprot/P00403) |
| H204 | H204 | H204 | Cu_A_ metal binding site | UniProt (http://www.uniprot.org/uniprot/P00403) |
|  | **(ii) MUSCLE alignment** | |  |  |
| **(i) Superimposition (Structure-based alignment)** | |  |  |  |
| **Profile alignment** | | |  |  |

**References:**

1. Marechal A, Meunier B, Lee D, Orengo C, Rich PR (2012) Yeast cytochrome c oxidase: a model system to study mitochondrial forms of the haem-copper oxidase superfamily. Biochim Biophys Acta 1817: 620-628.

2. Rahman S, Taanman JW, Cooper JM, Nelson I, Hargreaves I, et al. (1999) A missense mutation of cytochrome oxidase subunit II causes defective assembly and myopathy. Am J Hum Genet 65: 1030-1039.
