# Supplementary material for: Adaptation of the Mitochondrial Genome in Cephalopods: Enhancing Proton Translocation Channels and the Subunit Interactions
Source: PLoS One. 2015 Aug 18;10(8):e0135405. doi: 10.1371/journal.pone.0135405 (PMC4540416; doi:10.1371/journal.pone.0135405)
Supplement: S7 Table — The sites identified as positively selected by branch-site analyses (CODEML and MEME: p-value < 0.05) were mapped in the Cephalopoda COX3 protein sequence alignment (Cephalopoda COX3 dataset: obtained through the translation of the respective MUSCLE codon based CDS alignment; performed in SEAVIEW software version 4.4.0). (i) Then, we performed the superimposition (structure-based alignment) of the available COX3 X-ray crystal structure (Rhodobacter sphaeroides PDB: 1M56:C) with the corresponding COX3 3D structure of Octopus vulgaris (predicted in this study), using the PYMOL software version 1.5.0.4. Thus, we obtained a correspondence of the positively selected site numbering (assuming as reference the COX3 protein sequence of the Octopus vulgaris) to the COX3 sequence numbers of Rhodobacter sphaeroides species, which has described sites involved in proton coupling mechanisms (D-channel). (ii) We also performed a MUSCLE alignment (in the SEAVIEW software version 4.4.0) of the Cephalopoda COX3 dataset with its homolog from Homo sapiens. This approach allowed to establish a correspondence of described sites mutations related with diseases in humans, between Homo sapiens and cephalopods (e.g. Octopus vulgaris). Finally, we performed a profile alignment (using the GENEIOUS software version 5.6.7 profile align option) of the previous described alignments (i and ii), which allowed a correspondence of the sites among all the mentioned species. TREESAAP is mentioned when a site also presented amino acid properties positively selected (p-value < 0.001). (DOCX) [file pone.0135405.s011.docx]

**S7 Table. Homology analyses of the COX3 subunit.** The sites identified as positively selected by branch-site analyses (CODEML and MEME: p-value < 0.05) were mapped in the Cephalopoda COX3 protein sequence alignment (Cephalopoda COX3 dataset: obtained through the translation of the respective MUSCLE codon based CDS alignment; performed in SEAVIEW software version 4.4.0). (i) Then, we performed the superimposition (structure-based alignment) of the available COX3 X-ray crystal structure (*Rhodobacter sphaeroides* PDB: 1M56:C) with the corresponding COX3 3D structure of *Octopus vulgaris* (predicted in this study), using the PYMOL software version 1.5.0.4. Thus, we obtained a correspondence of the positively selected site numbering (assuming as reference the COX3 protein sequence of the *Octopus vulgaris*) to the COX3 sequence numbers of *Rhodobacter sphaeroides* species, which has described sites involved in proton coupling mechanisms (D-channel). (ii) We also performed a MUSCLE alignment (in the SEAVIEW software version 4.4.0) of the Cephalopoda COX3 dataset with its homolog from *Homo sapiens*. This approach allowed to establish a correspondence of described sites mutations related with diseases in humans, between *Homo sapiens* and cephalopods (e.g. *Octopus vulgaris*). Finally, we performed a profile alignment (using the GENEIOUS software version 5.6.7 profile align option) of the previous described alignments (i and ii), which allowed a correspondence of the sites among all the mentioned species. TREESAAP is mentioned when a site also presented amino acid properties positively selected (p-value < 0.001).

|  | **Cephalopoda COX3 dataset** |  |  |  |
| --- | --- | --- | --- | --- |
| ***Rhodobacter sphaeroides* (Bacterium PDB: 1M56:C)** | ***Octopus vulgaris* (Common octopus - NC_006353)** | ***Homo sapiens*  (Human - P00414)** | **Features** | **References** |
| H7 | N4 | H6 | Entrance of the D-channel; TREESAAP | [1] and This study |
| H10 | H7 | H9 | Entrance of the D-channel | [1] |
| I11 | L8 | M10 | Entrance of the D-channel | [1] |
| L12 | V9 | V11 | Entrance of the D-channel | [1] |
| W59 | W56 | W58STOP | Human related disease | [2] |
| L77 | N75 | K77 | CODEML and TREESAAP | This study |
| G78 | G76 | G78S | Human related disease | [1] |
| E112 | I110 | L112 | MEME | This study |
| D163 | S156 | Q158 | CODEML | This study |
| A205 | A198 | A200T | Human related disease | [3] |
| R229 | M222 | M224 | CODEML and TREESAAP | This study |
| W254 | W247 | W249STOP | Human related disease | [3] |
| F256 | F249 | F251L | Human related disease | [3] |
|  | **(ii) MUSCLE alignment** | |  |  |
| **(i) Superimposition (Structure-based alignment)** | |  |  |  |
| **Profile alignment** | | |  |  |

**References:**

1. Alnajjar KS, Hosler J, Prochaska L (2014) Role of the N-terminus of subunit III in proton uptake in cytochrome c oxidase of Rhodobacter sphaeroides. Biochemistry 53: 496-504.

2. Horvath R, Scharfe C, Hoeltzenbein M, Do BH, Schroder C, et al. (2002) Childhood onset mitochondrial myopathy and lactic acidosis caused by a stop mutation in the mitochondrial cytochrome c oxidase III gene. J Med Genet 39: 812-816.

3. Marechal A, Meunier B, Lee D, Orengo C, Rich PR (2012) Yeast cytochrome c oxidase: a model system to study mitochondrial forms of the haem-copper oxidase superfamily. Biochim Biophys Acta 1817: 620-628.
