# Supplementary material for: Adaptation of the Mitochondrial Genome in Cephalopods: Enhancing Proton Translocation Channels and the Subunit Interactions
Source: PLoS One. 2015 Aug 18;10(8):e0135405. doi: 10.1371/journal.pone.0135405 (PMC4540416; doi:10.1371/journal.pone.0135405)
Supplement: S8 Table — (DOCX) [file pone.0135405.s012.docx]

| **Gene** | **Model** | ***np*** | **Parameter estimates** | ***k*** | ***lnL*** | **Model comparison** | ***2∆lnL (LRT)*** | ***df*** | ***p-value*** |
| --- | --- | --- | --- | --- | --- | --- | --- | --- | --- |
| *atp6* | M0 | 33 | ω0 = 0.01267 | 1.68378 | -6303.32022 | M0 vs M3 | 425.29789 | 4 | 0.00 |
|  | M3 | 37 | ω0 = 0.00054 p0 = 0.40284 ω1 = 0.00869 p1 = 0.41092 ω2 = 0.06488 p2 = 0.186224 | 1.80378 | -6090.67127 |  |  |  |  |
|  | M1 | 34 | ω0 = 0.01385 p0 = 0.98187 ω1 = 1.00000 p1 = 0.01813 | 1.73644 | -6287.20706 | M1a vs M2a | 0.00000 | 2 | 1.00 |
|  | M2 | 36 | ω0 = 0.01385 p0 = 0.98187 ω1 = 1.00000 p1 = 0.00677 ω2 = 1.00000 p2 = 0.01135 | 1.73644 | -6287.20706 |  |  |  |  |
|  | M7 | 34 | p = 0.33138 q = 18.51467 | 1.81148 | -6093.75432 | M7 vs M8 | 0.00497 | 2 | 1.00 |
|  | M8 | 36 | p0 = 0.99999 p = 0.33138 q = 18.51462 (p1 = 0.00001) ω = 1.00000 | 1.81147 | -6093.75681 |  |  |  |  |
| *atp8* | M0 | 33 | ω0 = 0.07798 | 1.65014 | -1541.55792 | M0 vs M3 | 137.00135 | 4 | 0.00 |
|  | M3 | 37 | ω0 = 0.00263 p0 = 0.35527 ω1 = 0.03335 p1 = 0.31133 ω2 = 0.21048 p2 = 0.33340 | 1.74088 | -1473.05724 |  |  |  |  |
|  | M1 | 34 | ω0 = 0.05202 p0 = 0.68389 ω1 = 1.00000 p1 = 0.31611 | 2.85854 | -1511.83027 | M1a vs M2a | 0.00000 | 2 | 1.00 |
|  | M2 | 36 | ω0 = 0.05202 p0 = 0.68389 ω1 = 1.00000 p1 = 0.00509 ω2 = 1.00000 p2 = 0.31103 | 2.85854 | -1511.83027 |  |  |  |  |
|  | M7 | 34 | p = 0.35166 q = 3.63084 | 1.77388 | -1472.51833 | M7 vs M8 | 0.00078 | 2 | 1.00 |
|  | M8 | 36 | p0 = 0.99999 p = 0.35166 q = 3.63087 (p1 = 0.00001) ω = 1.00000 | 1.77389 | -1472.51872 |  |  |  |  |
| *cox1* | M0 | 33 | ω0 = 0.00575 | 2.70906 | -10749.21248 | M0 vs M3 | 226.35108 | 4 | 0.00 |
|  | M3 | 37 | ω0 = 0.00083 p0 = 0.75032 ω1 = 0.01748 p1 = 0.22039 ω2 = 0.04347 p2 = 0.02930 | 2.69921 | -10636.03694 |  |  |  |  |
|  | M1 | 34 | ω0 = 0.00575 p0 = 0.99999 ω1 = 1.00000 p1 = 0.00001 | 2.70906 | -10749.21755 | M1a vs M2a | 0.01013 | 2 | 0.99 |
|  | M2 | 36 | ω0 = 0.00575 p0 = 1.00000 ω1 = 1.00000 p1 = 0.00000 ω2 = 180.36288 p2 = 0.00000 | 2.70905 | -10749.21248 |  |  |  |  |
|  | M7 | 34 | p = 0.00500 q = 0.15041 | 2.67213 | -10729.56744 | M7 vs M8 | 0.01020 | 2 | 0.99 |
|  | M8 | 36 | p0 = 0.99999 p = 0.24230 q = 37.67988 (p1 = 0.00001) ω = 2.18127 | 2.69720 | -10636.51739 |  |  |  |  |
| *cox2* | M0 | 33 | ω0 = 0.01394 | 2.21349 | -5342.01011 | M0 vs M3 | 274.09334 | 4 | 0.00 |
|  | M3 | 37 | ω0 = 0.00021 p0 = 0.50402 ω1 = 0.01648 p1 = 0.42655 ω2 = 0.10685 p2 = 0.06943 | 2.41071 | -5204.96344 |  |  |  |  |
|  | M1 | 34 | ω0 = 0.01301 p0 = 0.99096 ω1 = 1.00000 p1 = 0.00904 | 2.32343 | -5331.25438 | M1a vs M2a | 0.00000 | 2 | 1.00 |
|  | M2 | 36 | ω0 = 0.01301 p0 = 0.99096 ω1 = 1.00000 p1 = 0.00904 ω2 = 3.63225 p2 = 0.00000 | 2.32343 | -5331.25438 |  |  |  |  |
|  | M7 | 34 | p = 0.27410 q = 15.37467 | 2.34549 | -5211.33423 | M7 vs M8 | 0.00448 | 2 | 1.00 |
|  | M8 | 36 | p0 = 0.99999 p = 0.27410 q = 15.37475 (p1 = 0.00001) ω = 1.00000 | 2.34550 | -5211.33648 |  |  |  |  |
| *cox3* | M0 | 33 | ω0 = 0.02210 | 2.11058 | -6757.55321 | M0 vs M3 | 448.50585 | 4 | 0.00 |
|  | M3 | 37 | ω0 = 0.00196 p0 = 0.65314 ω1 = 0.04727 p1 = 0.29355 ω2 = 0.15266 p2 = 0.05330 | 2.17575 | -6533.30028 |  |  |  |  |
|  | M1 | 34 | ω0 = 0.01913 p0 = 0.97554 ω1 = 1.00000 p1 = 0.02446 | 2.32287 | -6728.99078 | M1a vs M2a | 0.00001 | 2 | 1.00 |
|  | M2 | 36 | ω0 = 0.01913 p0 = 0.97554 ω1 = 1.00000 p1 = 0.02446 ω2 = 131.26299 p2 = 0.00000 | 2.32287 | -6728.99078 |  |  |  |  |
|  | M7 | 34 | p = 0.25913 q = 9.91041 | 2.17730 | -6535.72718 | M7 vs M8 | 0.00473 | 2 | 1.00 |
|  | M8 | 36 | p0 = 0.99999 p = 0.25913 q = 9.91060 (p1 = 0.00001) ω = 1.00000 | 2.17731 | -6535.72955 |  |  |  |  |
| *cytb* | M0 | 33 | ω0 = 0.01230 | 2.34869 | -8710.74199 | M0 vs M3 | 470.43657 | 4 | 0.00 |
|  | M3 | 37 | ω0 = 0.00054 p0 = 0.48775 ω1 = 0.01121 p1 = 0.34447 ω2 = 0.05238 p2 = 0.16777 | 2.69987 | -8475.52371 |  |  |  |  |
|  | M1 | 34 | ω0 = 0.01204 p0 = 0.99244 ω1 = 1.00000 p1 = 0.00756 | 2.37861 | -8707.42389 | M1a vs M2a | 0.00000 | 2 | 1.00 |
|  | M2 | 36 | ω0 = 0.01204 p0 = 0.99244 ω1 = 1.00000 p1 = 0.00108 ω2 = 1.00000 p2 = 0.00648 | 2.37861 | -8707.42389 |  |  |  |  |
|  | M7 | 34 | p = 0.32470 q = 22.68479 | 2.72212 | -8475.20833 | M7 vs M8 | 0.00758 | 2 | 1.00 |
|  | M8 | 36 | p0 = 0.99999 p = 0.32470 q = 22.68479 (p1 = 0.00001) ω = 14.24926 | 2.72212 | -8475.21212 |  |  |  |  |

**S8 Table. Likelihood ratio tests for PAML (CODEML) site models in 13 mitochondrial genes of the Cephalopoda dataset.**

| *nd1* | M0 | 33 | ω0 = 0.01292 | 2.37184 | -8117.08002 | M0 vs M3 | 622.97180 | 4 | 0.00 |
| --- | --- | --- | --- | --- | --- | --- | --- | --- | --- |
|  | M3 | 37 | ω0 = 0.00000 p0 = 0.00000 ω1 = 0.00136 p1 = 0.65053 ω2 = 0.03502 p2 = 0.34947 | 2.89417 | -7830.88887 |  |  |  |  |
|  | M1 | 34 | ω0 = 0.01165 p0 = 0.97505 ω1 = 1.00000 p1 = 0.02495 | 2.55483 | -8098.44354 | M1a vs M2a | 0.00000 | 2 | 1.00 |
|  | M2 | 36 | ω0 = 0.01165 p0 = 0.97505 ω1 = 1.00000 p1 = 0.02495 ω2 = 16.95645 p2 = 0.00000 | 2.55482 | -8098.44354 |  |  |  |  |
|  | M7 | 34 | p = 0.27813 q = 17.53422 | 3.05025 | -7807.96027 | M7 vs M8 | 0.00634 | 2 | 1.00 |
|  | M8 | 36 | p0 = 0.99999 p = 0.27813 q = 17.53422 (p1 = 0.00001) ω = 1.00000 | 3.05023 | -7807.96344 |  |  |  |  |
| *nd2* | M0 | 33 | ω0 = 0.01641 | 1.45303 | -11005.08008 | M0 vs M3 | 623.30410 | 4 | 0.00 |
|  | M3 | 37 | ω0 = 0.00265 p0 = 0.45478 ω1 = 0.02221 p1 = 0.45155 ω2 = 0.08933 p2 = 0.09367 | 1.45454 | -10693.42802 |  |  |  |  |
|  | M1 | 34 | ω0 = 0.01549 p0 = 0.95844 ω1 = 1.00000 p1 = 0.04156 | 1.51751 | -10940.87876 | M1a vs M2a | 0.00000 | 2 | 1.00 |
|  | M2 | 36 | ω0 = 0.01549 p0 = 0.95844 ω1 = 1.00000 p1 = 0.04156 ω2 = 311.45614 p2 = 0.00000 | 1.51751 | -10940.87876 |  |  |  |  |
|  | M7 | 34 | p = 0.58246 q = 26.89268 | 1.47472 | -10688.16903 | M7 vs M8 | 0.00346 | 2 | 1.00 |
|  | M8 | 36 | p0 = 0.99999 p = 0.58247 q = 26.89427 (p1 = 0.00001) ω = 1.00000 | 1.47471 | -10688.17076 |  |  |  |  |
| *nd3* | M0 | 33 | ω0 = 0.02242 | 1.74714 | -3416.84184 | M0 vs M3 | 235.56951 | 4 | 0.00 |
|  | M3 | 37 | ω0 = 0.00033 p0 = 0.34243 ω1 = 0.00748 p1 = 0.27317 ω2 = 0.05432 p2 = 0.38440 | 2.04131 | -3299.05708 |  |  |  |  |
|  | M1 | 34 | ω0 = 0.02046 p0 = 0.97624 ω1 = 1.00000 p1 = 0.02376 | 1.75216 | -3411.72713 | M1a vs M2a | 0.00000 | 2 | 1.00 |
|  | M2 | 36 | ω0 = 0.02046 p0 = 0.97624 ω1 = 1.00000 p1 = 0.02376 ω2 = 33.74599 p2 = 0.00000 | 1.75216 | -3411.72713 |  |  |  |  |
|  | M7 | 34 | p = 0.35352 q = 14.00218 | 2.03165 | -3300.30216 | M7 vs M8 | 0.00224 | 2 | 1.00 |
|  | M8 | 36 | p0 = 0.99999 p = 0.35352 q = 14.00226 (p1 = 0.00001) ω = 1.00000 | 2.03166 | -3300.30327 |  |  |  |  |
| *nd4* | M0 | 33 | ω0 = 0.02377 | 2.62307 | -14329.01336 | M0 vs M3 | 1227.59697 | 4 | 0.00 |
|  | M3 | 37 | ω0 = 0.00203 p0 = 0.44203 ω1 = 0.02597 p1 = 0.43433 ω2 = 0.12151 p2 = 0.12364 | 3.33951 | -13715.21488 |  |  |  |  |
|  | M1 | 34 | ω0 = 0.02356 p0 = 0.91690 ω1 = 1.00000 p1 = 0.08310 | 3.08217 | -14167.72127 | M1a vs M2a | 0.00000 | 2 | 1.00 |
|  | M2 | 36 | ω0 = 0.02356 p0 = 0.91690 ω1 = 1.00000 p1 = 0.05149 ω2 = 1.00000 p2 = 0.03160 | 3.08217 | -14167.72127 |  |  |  |  |
|  | M7 | 34 | p = 0.41541 q = 13.37775 | 3.38953 | -13701.95349 | M7 vs M8 | 0.42324 | 2 | 0.81 |
|  | M8 | 36 | p0 = 0.99839 p = 0.42002 q = 13.77140 (p1 = 0.00161) ω = 1.00000 | 3.39137 | -13701.74187 |  |  |  |  |
| *nd4l* | M0 | 33 | ω0 = 0.02213 | 1.89552 | -2698.01882 | M0 vs M3 | 103.63376 | 4 | 0.00 |
|  | M3 | 37 | ω0 = 0.00747 p0 = 0.61626 ω1 = 0.04119 p1 = 0.36064 ω2 = 0.19914 p2 = 0.02310 | 1.92142 | -2646.20194 |  |  |  |  |
|  | M1 | 34 | ω0 = 0.02089 p0 = 0.97994 ω1 = 1.00000 p1 = 0.02006 | 1.86396 | -2687.19107 | M1a vs M2a | 0.00000 | 2 | 1.00 |
|  | M2 | 36 | ω0 = 0.02089 p0 = 0.97994 ω1 = 1.00000 p1 = 0.00175 ω2 = 1.00000 p2 = 0.01831 | 1.86396 | -2687.19107 |  |  |  |  |
|  | M7 | 34 | p = 0.81944 q = 31.67080 | 2.00833 | -2644.29529 | M7 vs M8 | 0.00096 | 2 | 1.00 |
|  | M8 | 36 | p0 = 0.99999 p = 0.81948 q = 31.67349 (p1 = 0.00001) ω = 1.00000 | 2.00828 | -2644.29577 |  |  |  |  |
| *nd5* | M0 | 33 | ω0 = 0.01642 | 2.14460 | -17087.92856 | M0 vs M3 | 1654.07269 | 4 | 0.00 |
|  | M3 | 37 | ω0 = 0.00070 p0 = 0.40475 ω1 = 0.01163 p1 = 0.35240 ω2 = 0.05878 p2 = 0.24285 | 2.74944 | -16260.89221 |  |  |  |  |
|  | M1 | 34 | ω0 = 0.01678 p0 = 0.95536 ω1 = 1.00000 p1 = 0.04464 | 2.22217 | -16970.10826 | M1a vs M2a | 0.00000 | 2 | 1.00 |
|  | M2 | 36 | ω0 = 0.01678 p0 = 0.95536 ω1 = 1.00000 p1 = 0.01398 ω2 = 1.00000 p2 = 0.03066 | 2.22217 | -16970.10826 |  |  |  |  |
|  | M7 | 34 | p = 0.36001 q = 16.14858 | 2.76518 | -16240.30404 | M7 vs M8 | 1.95881 | 2 | 0.38 |
|  | M8 | 36 | p0 = 0.99701 p = 0.36729 q = 17.24006 (p1 = 0.00299) ω = 1.00000 | 2.77045 | -16239.32463 |  |  |  |  |

| *nd6* | M0 | 33 | ω0 = 0.00916 | 3.61057 | -5142.07129 | M0 vs M3 | 369.81189 | 4 | 0.00 |
| --- | --- | --- | --- | --- | --- | --- | --- | --- | --- |
|  | M3 | 37 | ω0 = 0.00034 p0 = 0.25230 ω1 = 0.00770 p1 = 0.41628 ω2 = 0.03847 p2 = 0.33143 | 3.76671 | -4957.16535 |  |  |  |  |
|  | M1 | 34 | ω0 = 0.00950 p0 = 0.97649 ω1 = 1.00000 p1 = 0.02351 | 3.77173 | -5132.62696 | M1a vs M2a | 0.08723 | 2 | 0.96 |
|  | M2 | 36 | ω0 = 0.00934 p0 = 0.97790 ω1 = 1.00000 p1 = 0.00161 ω2 = 1.00000 p2 = 0.02049 | 3.74794 | -5132.58334 |  |  |  |  |
|  | M7 | 34 | p = 0.54512 q = 29.51217 | 3.76704 | -4952.76909 | M7 vs M8 | 0.00462 | 2 | 1.00 |
|  | M8 | 36 | p0 = 0.99999 p = 0.54512 q = 29.51190 (p1 = 0.00001) ω = 1.00000 | 3.76704 | -4952.77140 |  |  |  |  |
