# Supplementary material for: Adaptation of the Mitochondrial Genome in Cephalopods: Enhancing Proton Translocation Channels and the Subunit Interactions
Source: PLoS One. 2015 Aug 18;10(8):e0135405. doi: 10.1371/journal.pone.0135405 (PMC4540416; doi:10.1371/journal.pone.0135405)
Supplement: S9 Table — A to H indicate foreground-lineages selected according with the hypotheses displayed in Fig 3. Values are the ϖ for the foreground-lineages tested (A to H). The colours indicate the final hypothesis decision, according to the LRT between the null and the alternate model likelihoods, considering as significant a p-value < 0.05. (DOCX) [file pone.0135405.s013.docx]

**S9 Table. Branch model test (CODEML) for the foreground cephalopod lineages.** A to H indicate foreground-lineages selected according with the hypotheses displayed in Fig 3. Values are the ϖ for the foreground-lineages tested (A to H). The colours indicate the final hypothesis decision, according to the LRT between the null and the alternate model likelihoods, considering as significant a p-value < 0.05.

|  |  | Cephalopoda foreground branch tested | | | | | | | |
| --- | --- | --- | --- | --- | --- | --- | --- | --- | --- |
|  |  | **A** | **B** | **C** | **D** | **E** | **F** | **G** | **H** |
| Gene CDS | **atp6** | 0.013 | 0.013 | 0.013 | 0.013 | 0.013 | 0.013 | 0.013 | 0.013 |
|  | **atp8** | 0.078 | 0.078 | 0.078 | 0.078 | 0.078 | 0.078 | 0.078 | 0.078 |
|  | **cox1** | 0.006 | 0.006 | 1.000 | 1.000 | 1.000 | 1.000 | 1.000 | 0.036 |
|  | **cox2** | 1.000 | 1.000 | 1.000 | 1.000 | 0.014 | 0.014 | 0.014 | 0.014 |
|  | **cox3** | 1.000 | 1.000 | 1.000 | 1.000 | 0.022 | 1.000 | 0.022 | 0.022 |
|  | **cytb** | 0.012 | 1.000 | 0.012 | 1.000 | 0.012 | 0.012 | 0.012 | 0.002 |
|  | **nd1** | 0.013 | 1.000 | 0.013 | 0.013 | 0.013 | 0.013 | 0.013 | 0.013 |
|  | **nd2** | 0.016 | 0.016 | 0.016 | 0.016 | 0.016 | 0.016 | 0.016 | 0.016 |
|  | **nd3** | 0.022 | 0.022 | 0.022 | 0.022 | 0.022 | 0.022 | 0.022 | 0.022 |
|  | **nd4** | 0.024 | 0.024 | 0.024 | 0.024 | 0.024 | 0.024 | 0.024 | 0.024 |
|  | **nd4l** | 0.022 | 0.022 | 0.022 | 0.022 | 0.022 | 0.022 | 0.022 | 0.022 |
|  | **nd5** | 0.016 | 0.016 | 1.000 | 1.000 | 0.016 | 0.016 | 0.016 | 0.016 |
|  | **nd6** | 0.009 | 0.009 | 0.009 | 0.009 | 0.009 | 0.009 | 0.009 | 0.009 |

**TEST 1: Null (one-ratio) vs. Alternate (unconstrained two-ratio)**

**TEST 2: Null (constrained two-ratio) vs. Alternate (unconstrained two-ratio)**

**TEST RESULTS:**

**Null (one-ratio) model selected -** all branches are supposed to evolve at a same rate (ϖF = ϖB).

**Alternate (unconstrained two-ratio) model selected** **-** the foreground (F) branch obtained ϖF<1 and a different evolutionary rate relative to other branches (B) (ϖF≠ϖB).

**Null (constrained two-ratio) model selected -** the foreground branch obtained ϖF=1, relaxed selection constraints.

**Numbers -** foreground average omega ratio (ϖF) value, allowed to vary among branches.
