# Supplementary material for: Adaptation of the Mitochondrial Genome in Cephalopods: Enhancing Proton Translocation Channels and the Subunit Interactions
Source: PLoS One. 2015 Aug 18;10(8):e0135405. doi: 10.1371/journal.pone.0135405 (PMC4540416; doi:10.1371/journal.pone.0135405)
Supplement: S10 Table — A to H indicate foreground-lineages selected according with the hypotheses displayed in Fig 3. Values are the ω for the foreground-lineages tested (A to H). The colours indicate the final hypothesis decision, according to the LRT between the null and the alternate model likelihoods, considering as significant a p-value < 0.05. (DOCX) [file pone.0135405.s014.docx]

**S10 Table. Branch-site model test (CODEML) for the foreground cephalopod lineages.** A to H indicate foreground-lineages selected according with the hypotheses displayed in Fig 3. Values are the ω for the foreground-lineages tested (A to H). The colours indicate the final hypothesis decision, according to the LRT between the null and the alternate model likelihoods, considering as significant a p-value < 0.05.

|  |  | Cephalopoda foreground branch tested | | | | | | | |
| --- | --- | --- | --- | --- | --- | --- | --- | --- | --- |
|  |  | **A** | **B** | **C** | **D** | **E** | **F** | **G** | **H** |
| Gene CDS | **atp6** | 1.000 | 1.000 | 1.000 | 1.000 | 1.000 | 1.000 | 1.000 | 1.000 |
|  | **atp8** | 1.000 | 1.000 | 1.000 | 1.000 | 1.000 | 1.000 | 1.000 | 1.000 |
|  | **cox1** | 1.000 | 1.000 | 1.000 | 1.000 | 1.000 | 1.000 | 1.000 | 1.000 |
|  | **cox2** | 1.000 | 1.000 | 999.000 | 1.000 | 1.000 | 1.000 | 999.000 | 1.000 |
|  | **cox3** | 1.000 | 1.000 | 1.000 | 1.000 | 54.175 | 999.000 | 1.000 | 1.000 |
|  | **cytb** | 1.000 | 1.000 | 1.000 | 1.000 | 1.000 | 1.000 | 1.000 | 1.000 |
|  | **nd1** | 1.000 | 7.048 | 1.000 | 1.000 | 1.000 | 1.000 | 1.000 | 1.000 |
|  | **nd2** | 1.000 | 1.000 | 1.000 | 1.000 | 1.000 | 1.000 | 999.000 | 1.000 |
|  | **nd3** | 1.000 | 1.000 | 1.000 | 1.000 | 1.000 | 1.000 | 1.000 | 1.000 |
|  | **nd4** | 1.000 | 1.000 | 1.000 | 1.000 | 1.000 | 96.835 | 999.000 | 11.176 |
|  | **nd4l** | 1.000 | 1.000 | 1.000 | 1.000 | 1.000 | 1.000 | 1.000 | 1.000 |
|  | **nd5** | 1.000 | 1.000 | 999.000 | 1.000 | 18.071 | 1.000 | 999.000 | 1.000 |
|  | **nd6** | 1.000 | 1.000 | 1.000 | 1.000 | 1.000 | 1.000 | 999.000 | 1.000 |

**TEST: Null vs. Alternate**

**TEST RESULTS:**

**Null model selected -** the foreground branch was constrained to ω_F_=1.

**Alternate model selected -** foreground branch obtained ω_F_≥1.

**Numbers –** foreground omega ratio (ωF) ratio, allowed to vary among branches and sites. An ω=999.000 indicates dS=0, hence ω is undefined.
